# Phage display enables machine learning discovery of cancer antigen–specific TCRs

**DOI:** 10.1126/sciadv.ads5589

**Published:** 2025-06-11

**Authors:** Giancarlo Croce, Rachid Lani, Delphine Tardivon, Sara Bobisse, Mariastella de Tiani, Maiia Bragina, Marta A. S. Perez, Justine Michaux, Hui Song Pak, Alexandra Michel, Talita Gehret, Julien Schmidt, Philippe Guillame, Michal Bassani-Sternberg, Vincent Zoete, Alexandre Harari, Nathalie Rufer, Michael Hebeisen, Steven M. Dunn, David Gfeller

**Affiliations:** ^1^ Department of Oncology UNIL CHUV, Ludwig Institute for Cancer Research, University of Lausanne, Lausanne, Switzerland.; ^2^ Swiss Institute of Bioinformatics (SIB), Lausanne, Switzerland.; ^3^ Agora Cancer Research Centre, Lausanne, Switzerland.; ^4^ Swiss Cancer Center Leman (SCCL), Lausanne, Switzerland.; ^5^ Department of Oncology UNIL CHUV, Ludwig Institute for Cancer Research, University Hospital of Lausanne, Lausanne, Switzerland.

## Abstract

T cells targeting epitopes in infectious diseases or cancer play a central role in spontaneous and therapy-induced immune responses. Epitope recognition is mediated by the binding of the T cell receptor (TCR), and TCRs recognizing clinically relevant epitopes are promising for T cell–based therapies. Starting from a TCR targeting the cancer-testis antigen NY-ESO-1_157–165_ epitope, we built large phage display libraries of TCRs with randomized complementary determining region 3 of the β chain. The TCR libraries were panned against NY-ESO-1, which enabled us to collect thousands of epitope-specific TCR sequences. Leveraging these data, we trained a machine learning TCR-epitope interaction predictor and identified several epitope-specific TCRs from TCR repertoires. Cellular assays revealed that the predicted TCRs displayed activity toward NY-ESO-1 and no detectable cross-reactivity. Our work demonstrates how display technologies combined with TCR-epitope interaction predictors can effectively leverage large TCR repertoires for TCR discovery.

## Teaser

Phage display and machine learning join forces to identify cancer-specific TCRs.

## INTRODUCTION

T cells play a key role in infectious diseases and cancer immunotherapy (*1*–*3*). The T cell response is initiated by the binding of T cell receptors (TCRs) to specific peptides (referred to as epitopes) displayed on the surface of cells by major histocompatibility complex (MHC) molecules [also called human leukocyte antigens (HLAs)]. TCRs are heterodimer surface proteins composed of an α and a β chain. TCRs show extensive sequence diversity across different T cells and ~10^11^ T cells with distinct TCRs are constantly circulating in the human body (*4*–*6*). The TCR sequence diversity is achieved during the V(D)J recombination where a unique combination of the germline-encoded V and J segments—respectively, V, D, and J segments—are selected to form the α, respectively β, chain. Both chains undergo additional nucleotide insertions and deletions at the V(D)J junctions, thereby further increasing the diversity of TCR sequences. The V segments contain two complementarity-determining regions (CDR1 and CDR2), which primarily mediate contact with the MHC, and a third one [CDR3, located at V(D)J junctions], which is mainly involved in recognition of the epitope.

TCRs recognizing clinically relevant epitopes represent promising therapeutic agents for T cell–based immunotherapy. For instance, T cells enriched in TCRs recognizing cancer epitopes have been infused into patients to mount responses against different malignancies (*7*, *8*). TCRs recognizing specific epitopes also show promise for diagnostics because their presence in the TCR repertoire of a patient can inform clinicians of the past or present immunological status of this patient (*9*–*12*). From a more theoretical point of view, epitope-specific TCRs provide key information to characterize the specificity of TCR-epitope interactions (*13*, *14*).

Binding and activation assays have been widely used to isolate and sequence epitope-specific TCRs (*15*, *16*). These experiments typically involve in vitro stimulation of primary T cells from donors with the epitope of interest, followed by isolation and TCR sequencing of the epitope-specific T cells. Binding assays use individual peptide-MHC (pMHC) multimers (*17*–*19*) or multiplexed DNA barcoded pMHC multimers (*20*, *21*), coupled with flow cytometry to isolate epitope-specific T cells. Functional assays use specific markers, such as CD137, PD-1, or CD69, to identify epitope-specific T cells which are activated by epitope stimulation (*22*, *23*). These approaches have enabled researchers to sequence thousands of epitope-specific TCRs for several immunodominant epitopes restricted to frequent MHC alleles (*10*, *24*–*26*). However, the number of epitopes with enough TCRs for in-depth characterization of their specificity is still limited. For instance, less than 50 epitopes have more than 100 known full-length αβTCRs in public databases (*10*). The scarcity of data is especially pronounced for cancer epitopes, which are more challenging to profile in standard binding or functional T cell assays because the TCR repertoire of patients or donors typically contains only very few (if any) TCRs recognizing such epitopes (*27*). A prototypical example is the HLA-A*02:01 restricted NY-ESO-1_157–165_ epitope (*28*–*30*) (hereafter referred to as NY-ESO-1). NY-ESO-1 is a widely studied cancer-testis antigen with very low expression in normal, non-germline tissue, but it is aberrantly expressed in many tumors (*31*, *32*). NY-ESO-1 can elicit a T cell response and, therefore, represents a promising target for many T cell–based immunotherapies (*28*–*30*). NY-ESO-1–reactive T cells have been reported in the blood of some patients with metastatic melanoma (*33*, *34*) but are rare in other patients or in healthy donors. Now, less than 15 naturally occurring NY-ESO-1–specific TCRs are available in databases of epitope-specific TCRs like VDJdb (*27*). Such low numbers make it challenging to characterize the specificity of TCRs recognizing this epitope (*35*–*37*).

Naturally occurring TCRs targeting NY-ESO-1 are usually of low affinity (around 10 μM) (*38*–*40*), and several approaches have been used to design affinity-enhanced TCRs for therapeutic applications. These include using phage display to select large libraries of TCRs with randomized amino acids at specific positions by panning them against NY-ESO-1 (*41*–*46*). Alternatively, in silico protein engineering methods have also been used (*47*). Due to specific amino acid substitutions within the CDR1 and CDR2 regions of the α and β chains, these TCRs can have substantially higher affinities than naturally occurring TCRs, reaching the picomolar range (*42*, *43*). However, such TCRs carry an inherent risk of cross-reactivity, potentially targeting peptides displayed on MHCs other than the intended epitope (*44*, *48*–*51*). Additionally, high affinity can induce T cell dysfunction (*52*, *53*) and reactivity toward features of the MHC molecules in the absence of cognate peptides (*54*–*56*).

Machine learning predictors can help identify epitope-specific TCRs within vast pools of potential candidates, such as TCR repertoires. TCR-epitope interaction predictors range from distance-based classifiers (*13*, *57*, *58*) to machine learning or deep learning models (*10*, *24*, *59*–*67*). TCR-epitope interaction predictors have been shown to identify epitope-specific TCRs with reasonable accuracy if a large number of TCRs are available for a given epitope (~50 to 100 TCRs) (*10*, *68*) but struggle to achieve robust predictions for epitopes for which TCR data are scarce or absent (*16*, *67*, *69*, *70*). For these reasons, as of today, robust predictions can only be performed for a few dozens of epitopes (*16*, *71*).

In this study, we designed a phage display experiment to collect a large number of TCRs recognizing the NY-ESO-1 epitope. Integrating these data into a machine learning TCR-epitope interaction predictor enabled us to identify directly from native repertoires epitope-specific TCRs showing activity toward NY-ESO-1 and no detectable cross-reactivity.

## RESULTS

### Phage display reveals CDR3β binding motifs of TCRs specific for NY-ESO-1

**Fig. 1.**
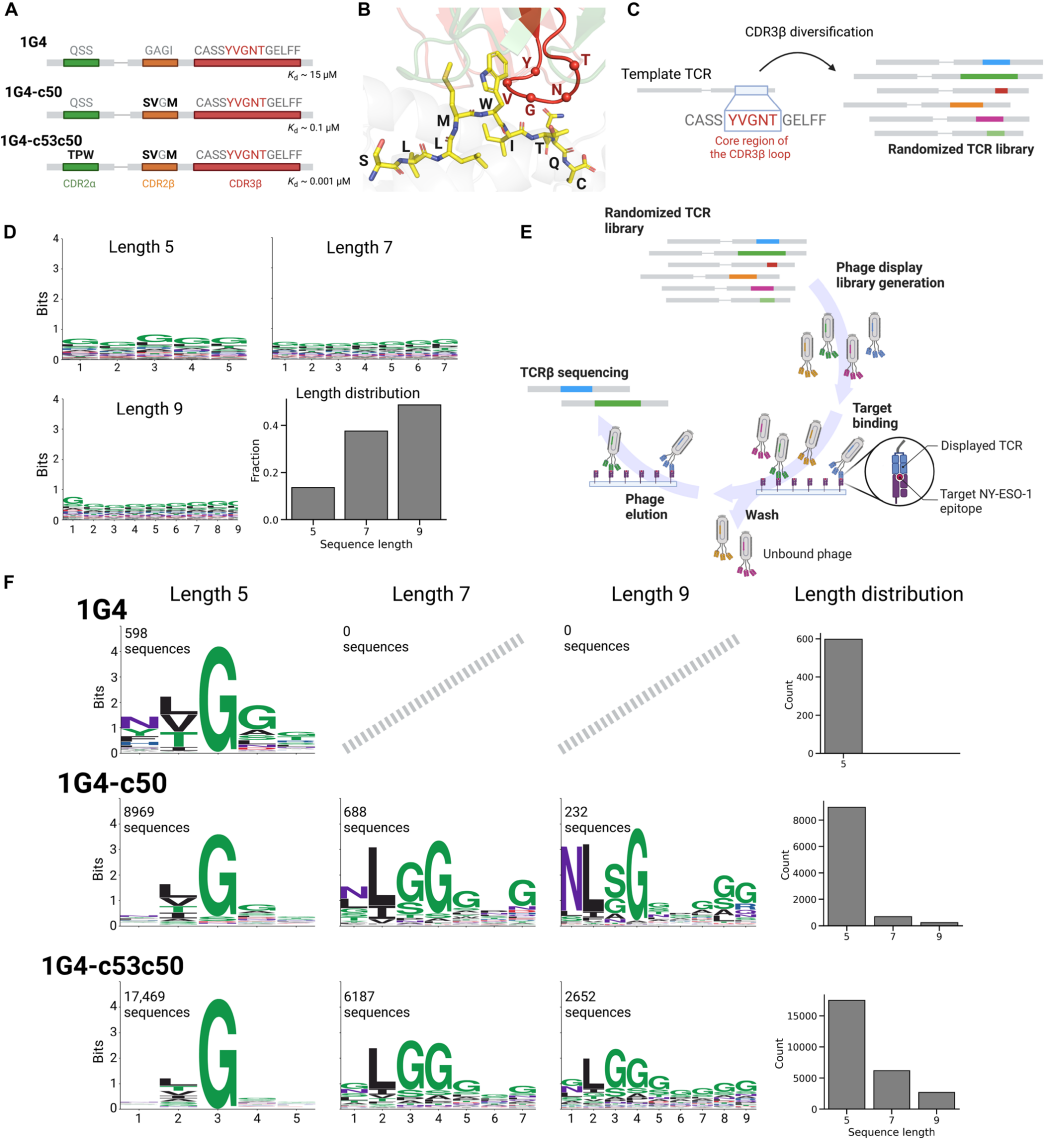
Phage display reveals CDR3β binding motifs of TCRs specific for NY-ESO-1. (**A**) Description of the three template TCRs used in the phage libraries. Amino acid substitutions of the 1G4-c50 and 1G4-c53c50 templates are highlighted in bold. The core region of the CDR3β (YVGNT) is highlighted in red. *K*_d_ is the dissociation constant, a measure of binding affinity. (**B**) Illustration of the 1G4 - NY-ESO-1 complex [PDB: 2BNR; (*78*)]. The α-carbons of the core region of the CDR3β loop are represented as spheres. (**C**) Schematic of the design of the randomized TCR libraries for the phage display experiments. The TCRs have random amino acid sequences of lengths 5, 7, and 9 in the core region of the CDR3β loops. (**D**) Sequence motifs and length distribution of the core region of CDR3β loops in input phage libraries. (**E**) Illustration of the phage display experiment. The randomized TCRs expressed in phages were panned against the NY-ESO-1 pMHC monomer and sequenced. (**F**) Motifs and length distributions of the core regions of the CDR3β loops resulting after selection of phage libraries and motif deconvolution. Created in BioRender. G. Croce (2025), https://BioRender.com/r19q929.

To decipher the specificity of TCRs recognizing the NY-ESO-1 epitope, we built large phage display libraries of TCRs with diversified CDR3β loops. As a template, we first used a naturally occurring TCR targeting the NY-ESO-1 epitope, known as 1G4, which was isolated from the TCR repertoire of a patient with melanoma (*32*). We further included a second and third template consisting of two affinity-enhanced TCRs, namely, the 1G4-c50 and 1G4-c53c50 TCRs (Fig. 1A and Table 1) (*43*). These two TCRs are characterized by amino acid substitutions in the CDR2 regions of 1G4, which interact with the MHC and substantially enhance the TCR affinity toward NY-ESO-1 (Fig. 1A) (*43*).

**Table 1.** Sequences of the 1G4, 1G4-c50, and 1G4-c53c50 template TCRs.

	Alpha chain [TRAV21*01/TRAJ6*01]			Beta chain [TRBV6-5*01/TRBJ2-2*01]		
Template TCR	CDR1	CDR2	CDR3	CDR1	CDR2	CDR3
1G4	DSAIYN	IQSSQRE	CAVRPTSGGSYIPTF	MNHEY	SVGAGI	CASSYVGNTGELFF
1G4-c50	DSAIYN	IQSSQRE	CAVRPTSGGSYIPTF	MNHEY	SV**SV**G**M**	CASSYVGNTGELFF
1G4-c53c50	DSAIYN	I**TPW**QRE	CAVRPTSGGSYIPTF	MNHEY	SV**SV**G**M**	CASSYVGNTGELFF

The core region of the CDR3β loop corresponds to the YVGNT five–amino acid sequence that directly interacts with the NY-ESO-1 epitope (Fig. 1B). This region was, therefore, selected for amino acid sequence and length diversification using a two-step polymerase chain reaction (PCR) process (Fig. 1C and fig. S1). The initial PCRs used a common forward primer and discrete reverse primers incorporating tails comprising different lengths of diversified trimer-defined codons. The chosen amino acid composition was designed to reflect that of naturally occurring TCRs (table S1). These individual length-variant PCR products were then used in a second PCR reaction to introduce an XhoI restriction site downstream of the diversified CDR3β to facilitate substitution cloning into the TCR-containing vector (see Methods, fig. S1, and table S2).

The randomized TCR library had a diversity larger than 10^8^ for each template, with randomized regions in the CDR3β loops of length 5, 7, and 9 (see Methods). While this diversity fully covers the theoretical sequence space for regions of length 5, it only captures a subset of the theoretical diversity for lengths 7 and 9 (table S3). To assess the quality of our input phage libraries, we sequenced them before any selection step (see Methods and data S1). Figure 1D shows the sequence motifs and the length distribution of the randomized regions merging the data for the three template TCRs (see fig. S3 for the data for each template). The slight enrichment in some amino acids (e.g., Gly) reflects the design of the input library (table S1). The N-terminal part (CASS) and C-terminal part (GELFF) of the CDR3β were not randomized because they do not directly interact with the epitope.

The TCR libraries were incorporated into phages and panned against the NY-ESO-1 pMHC monomer immobilized on magnetic beads (Fig. 1E). One round of panning was performed, incorporating different stringencies controlled by varying the number of wash cycles (one, three, and five washes) (see Methods). The enrichment of specific CDR3β sequences was assessed by sequencing the panned phage libraries after each wash cycle (see Methods and fig. S2).

CDR3β sequences obtained with one, three, and five washes were merged together as no notable differences were observed by varying the number of wash cycles (see Methods and fig. S2). A considerable number of unspecific TCRs are expected after one round of panning and washing the phage libraries. To filter out these putative contaminants, we used unsupervised motif deconvolution with MoDec (*72*) (see Methods). In most cases, one specific motif was found together with a second flat motif representing the background amino acid distribution of the input phage libraries (fig. S3 and data S2). To confirm that most TCRs in the flat motif identified by MoDec (i.e., putative contaminants) do not bind NY-ESO-1, we randomly selected five of them, transfected them in Jurkat cells (see Methods), and stained the cells with the NY-ESO-1 multimer (table S4). None of them could bind to NY-ESO-1 (fig. S4). The final binding motifs are reported in Fig. 1F separately for each template and each length of the core region of the CDR3β loops. With the 1G4 template, we already obtained several NY-ESO-1–specific TCRs (598 unique sequences). The 1G4-c50 and 1G4-c53c50 templates yielded a much higher number (9889 and 26,308 unique sequences, respectively). This aligns with expectations from their different intrinsic affinities (Fig. 1A). The sequence motifs displayed high similarities across all templates and lengths, with enrichment of hydrophobic amino acids (Leu, Ile, and Val) at position 2 and Gly at position 3 (Fig. 1F). With the 1G4 template, only CDR3β loops with randomized regions of five amino acids (i.e., 5-mers) could be retrieved in our pipeline (Fig. 1F). On the contrary, we obtained binding sequences for all three lengths with the 1G4-c50 and 1G4-c53c50 templates, albeit with a considerably higher number of 5-mers. Overall, this analysis reveals that reproducible CDR3β motifs can be obtained by expressing large libraries of TCRs with randomized CDR3β loops in phage and panning them with the NY-ESO-1 epitope.

### Integrating phage display data with machine learning tools enables robust predictions of NY-ESO-1–specific TCRs

**Fig. 2.**
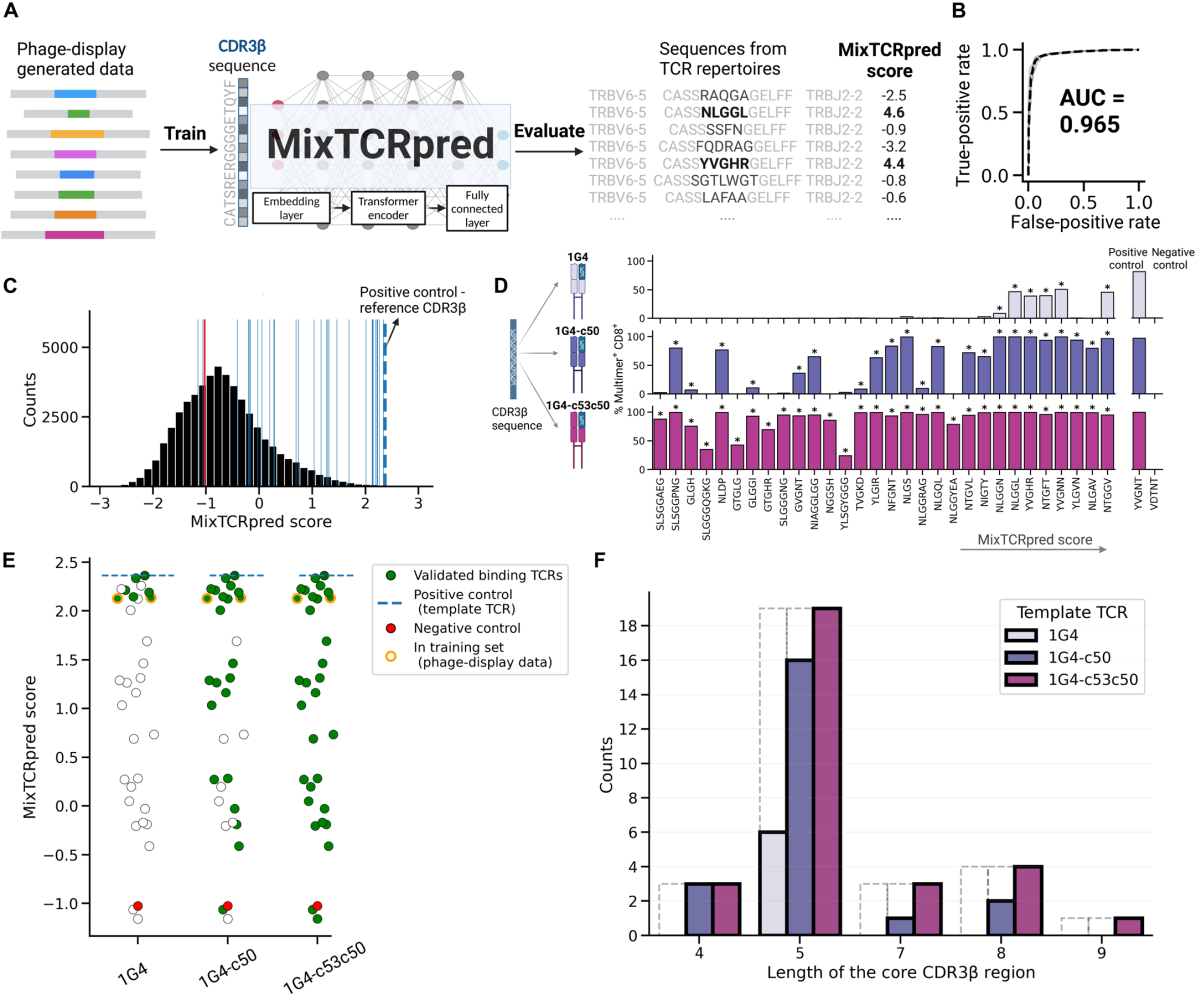
Integrating phage display data with machine learning tools enables robust predictions of NY-ESO-1–specific TCRs. (**A**) Illustration of training of MixTCRpred with CDR3β sequences obtained with the phage display screening, and evaluation of sequences from TCR repertoires of donors. (**B**) The receiver operating characteristic (ROC) curves obtained with a fivefold cross-validation based on the phage display data. The black dashed line is the mean ROC curve. (**C**) Distribution of the MixTCRpred scores of CDR3β sequences from TCR repertoires. The blue lines show the scores of the 30 CDR3β sequences selected for experimental testing. The dashed blue line shows the score of the reference CDR3β sequence CASS**YVGNT**GELFF. (**D**) Percentage of multimer^+^CD8^+^ cells among Jurkat cells transduced with each of the 30 TCRs selected in (C). TCRs are labeled on the basis of the sequence of the core region of CDR3β loops and ordered by the MixTCRpred scores. Stars indicate TCRs considered as NY-ESO-1 specific. (**E**) MixTCRpred scores of the TCRs with different CDR3β loops that could (green) or could not (white) be experimentally validated with the 1G4, 1G4-c50, and 1G4-c53c50 templates. The MixTCRpred scores of the positive control (the reference CDR3β sequence CASS**YVGNT**GELFF) and of the negative control (the CASS**VDTNT**GELFF sequence) are also shown. Points with an orange border represent TCRs that were also identified in the phage display screening and, therefore, part of the MixTCRpred training set. (**F**) Length of the core region of the CDR3β sequences that were tested (dashed lines) and validated (solid lines) for the three TCR templates. Created in BioRender. G. Croce (2025), https://BioRender.com/r28h762.

We leveraged the TCRβ sequences obtained in the phage display to train a TCR-epitope interaction predictor for NY-ESO-1. To this end, we used the MixTCRpred machine learning framework (*10*) and trained a specific model for this epitope (see Methods and Fig. 2A). MixTCRpred takes TCR sequences as inputs, processes them through an embedding layer, followed by a transformer encoder, and uses a fully connected layer for classification. This architecture outputs a prediction score that estimates the likelihood of TCR-epitope binding (Fig. 2A). As positives, we used all unique NY-ESO-1–specific CDR3β obtained with the naturally occurring template 1G4 (598 sequences). As negatives, we used CDR3β sequences from the randomized TCR libraries that did not bind NY-ESO-1 in the phage display experiment (see Methods). For quality control, we first performed a standard fivefold cross-validation. As expected from the highly specific motifs in Fig. 1F, we obtained high area under the receiver operating curve (AUC) values, with a mean AUC of 0.965 (Fig. 2B).

We next explored whether our MixTCRpred model could be used to identify NY-ESO-1–specific TCRs directly from native TCR repertoires. To this end, we first collected a large number of TCRβ sequences from TCR repertoires of unrelated donors (*73*). To be consistent with the design of our phage display libraries, we only included TCRβ with *TRBV6-5* and *TRBJ2-2* genes (see Methods). In total, we retrieved 29,867 TCRβ sequences, which were scored with our MixTCRpred model. The distribution of the MixTCRpred scores is shown in Fig. 2C. TCRβ with high scores are predicted to be NY-ESO-1 specific. The reference CDR3β sequence (CASS**YVGNT**GELFF) has a MixTCRpred score of 2.36, ranking as the top-scoring sequence (Fig. 2C).

To investigate and experimentally validate the potential of our in silico predictions, we selected 30 TCRβ with a broad range of MixTCRpred scores and including different lengths for the core region of the CDR3β loop (Fig. 2C and Table 2). We also included the reference CDR3β sequence (CASS**YVGNT**GELFF) as positive control and a randomly selected CDR3β (CASS**VDTNT**GELFF) having a score of −1.03 to use as negative control. All TCRs with each of the three templates (i.e., 1G4, 1G4-c50, and 1G4-c53c50) were tested for binding to the NY-ESO-1 epitope (SLLMWITQC) (Fig. 2D). To this end, RNA encoding each of the selected TCRs was synthesized and introduced into Jurkat cells via electroporation. Following overnight incubation, the TCR-transfected cells were interrogated for binding with NY-ESO-1-multimers (see Methods and Fig. 2D). An illustration of the results of the multimer staining is shown in fig. S5. A relatively small number of TCRs could be validated with the 1G4 template (6 of 30) with variable percentages of multimer^+^CD8^+^ Jurkar cells from the multimer staining experiment (Fig. 2D). The validated TCRs ranked among the top-scoring predictions with MixTCRpred. Two of them were also observed in the phage display data (i.e., were included in the MixTCRpred training set) (Fig. 2E). All six validated TCRs had core regions of the CDR3β loop of length 5 (Fig. 2F). Conversely, most (i.e., 22 of 30) TCRs with the 1G4-c50 template and all TCRs with the 1G4-c53c50 template were found to bind to NY-ESO-1 (Fig. 2, D and E). TCRs with CDR3β of multiple lengths could be validated with the affinity-enhanced templates (1G4-c50 and 1G4-c53c50), including some with lengths not included in the training set of our MixTCRpred model (Fig. 2F).

**Table 2.** CDR3β sequences selected for experimental validation, along with their MixTCRpred scores and the percentages of multimer^+^CD8^+^ T cells obtained from multimer-staining experiments. The experiments were performed using three template TCRs (1G4, 1G4-c50, and 1G4-c53c50). Reported values represent the averages of two independent replicates.

			% of multimer^+^CD8^+^ Jurkat cells from the multimer staining experiment		
CDR3β number	CDR3β sequence	MixTCRpred score	1G4	1G4-c50	1G4-c53c50
1	CASSNTGGVGELFF	2.34	45.8	96.5	95.1
2	CASSNLGAVGELFF	2.26	0.2	79.5	98.9
3	CASSYLGVNGELFF	2.23	0.4	93.8	99.3
4	CASSYVGNNGELFF	2.21	50.9	99.6	99.4
5	CASSNTGFTGELFF	2.19	39.9	93.7	95.8
6	CASSYVGHRGELFF	2.15	39.1	99.3	99.5
7	CASSNLGGLGELFF	2.13	46.8	99.6	99.4
8	CASSNLGGNGELFF	2.13	8.4	99.4	99.3
9	CASSNIGTYGELFF	2.12	2.6	65.1	99.0
10	CASSNTGVLGELFF	2.01	0.0	71.8	94.6
11	CASSNLGGYEAGELFF	1.69	0.3	0.4	78.8
12	CASSNLGQLGELFF	1.46	0.7	82.8	99.2
13	CASSNLGGRAGGELFF	1.31	0.4	9.8	96.0
141	CASSNLGSGELFF	1.29	2.7	99.3	99.3
15	CASSNFGNTGELFF	1.26	0.0	83.4	93.2
16	CASSYLGIRGELFF	1.16	0.3	63.1	99.3
17	CASSTVGKDGELFF	1.03	0.3	8.5	99.1
18	CASSYLSGYGGGGELFF	0.73	0.3	2.8	24.0
19	CASSNGGSHGELFF	0.69	0.0	0.1	85.8
20	CASSNIAGGLGGGELFF	0.28	0.3	64.9	94.9
21	CASSGVGNTGELFF	0.27	0.1	36.2	93.6
22	CASSSLGGGNGGELFF	0.20	0.2	1.4	95.0
23	CASSGTGHRGELFF	0.05	0.0	0.1	69.5
24	CASSGLGGIGELFF	−0.03	0.0	10.8	92.8
25	CASSGTGLGGELFF	−0.17	0.0	0.1	42.7
26	CASSNLDPGELFF	−0.19	0.2	76.7	99.2
27	CASSSLGGGQGKGGELFF	−0.20	0.3	0.1	34.9
28	CASSGLGHGELFF	−0.41	0.0	7.0	75.5
29	CASSSLSGGPNGGELFF	−1.06	0.2	80.1	99.3
30	CASSSLSGGAEGGELFF	−1.16	0.3	2.7	87.8
Positive control	CASSYVGNTGELFF	2.36	90.9	99.6	99.1
Negative control	CASSVDTNTGELFF	−1.03	0.0	0.0	0.0

To assess how using TCRs obtained with different templates in the phage libraries influenced the predictive power of MixTCRpred, we trained three template-specific models. Each model used as positives TCR sequence data obtained from the phage display screening with a specific template (see Methods and fig. S6). Despite the highly variable number of training data (598 positive with the 1G4 templates, 9889 with 1G4-c50, and 26,308 with 1G4-c53c50), the TCRs validated for NY-ESO-1 binding consistently ranked as top-scoring sequences for each of the three models (fig. S6). Additionally, we trained a combined model that incorporated all three datasets (1G4, 1G4-c50, and 1G4-c53c50), yielding consistent results (see fig. S6).

To explore whether predictions could be expanded to TCRs with TRBV and TRBJ different from those of the templates, we applied MixTCRpred to a dataset including 10 million TCRβs with diverse TRBV and TRBJ segments and selected the top six predicted ones for validation (table S5). Despite some similarity of the V/J sequences with those of the 1G4 template, we could not detect any binding (fig. S7). We further tested TCRs with the reference CDR3β (CASSYVGNTGELFF) and TRBJ, but other TRBV displaying high similarity to TRBV6-5*01, including all V segments of the TRBV6 family (table S6). Here again, we could not detect any binding (fig. S8). This demonstrates high specificity in V gene selection and supports our decision of allowing predictions only with the V and J segments used in the phage display experiment. Overall, this analysis demonstrates that the data obtained with the phage display pipeline can be effectively used to train a predictive model, which can then be used to identify NY-ESO-1–specific TCRβ sequences directly from TCR repertoires.

### Computational models trained on phage display data outperform other approaches for predictions of NY-ESO-1–specific TCRs

**Fig. 3.**
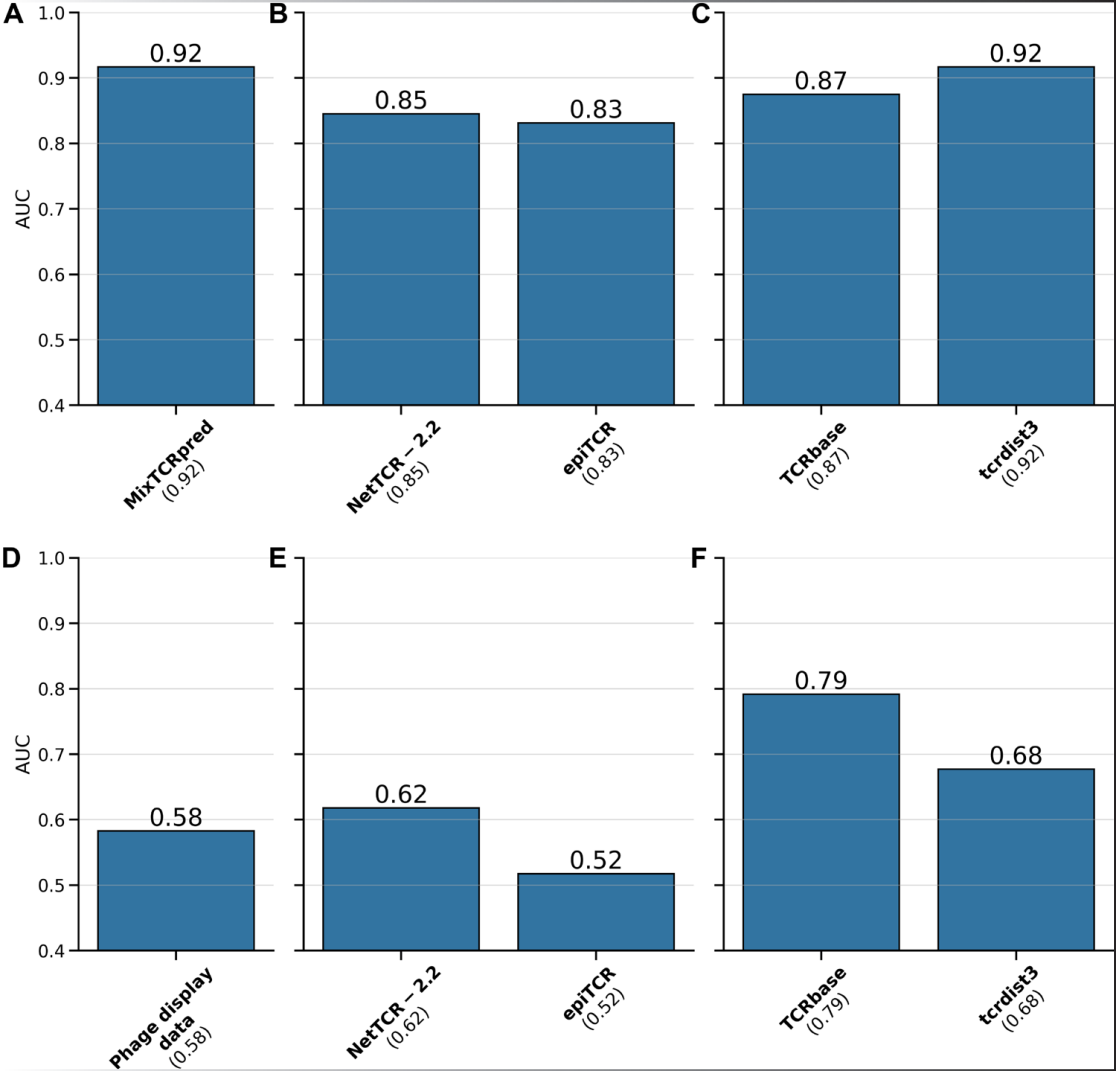
Computational models trained on phage display data outperforms other approaches for predictions of NY-ESO-1–specific TCRs. (**A**) AUC achieved by MixTCRpred trained on the phage display data generated with the 1G4 template TCRs. (**B**) AUCs obtained with two deep learning predictors including the data generated with the phage display in their training sets. (**C**) AUCs achieved with two distance-based predictors including the data generated with the phage display in their training sets. (**D**) AUC obtained by looking for an exact match of the TCRs in the data generated by phage display. (**E**) AUCs obtained with two pretrained deep learning predictors that do not include the data generated with the phage display in their training sets. (**F**) AUCs achieved with two distance-based models by computing sequence similarity to the reference CDR3β (CASS**YVGNT**GELFF). Created in BioRender. G. Croce (2025), https://BioRender.com/h07r133.

We next compared our strategy for identifying NY-ESO-1–specific TCRs with other approaches. To this end, we capitalized on the 30 experimentally tested TCRs in Fig. 2D with the 1G4 template (i.e., 6 positives and 24 negatives) and on the fact that they span a large range of MixTCRpred scores (i.e., were not restricted to the top scoring TCRs). MixTCRpred trained on the phage display data generated with the 1G4 template (598 NY-ESO-1–specific TCRs) achieved an AUC of 0.92 (Fig. 3A). We next used such data to retrain two deep learning TCR-epitope interaction predictors [NetTCR2.2 (*68*) and epiTCR (*74*), Fig. 3B] and two distance-based models [TCRbase (*57*) and tcrdist3 (*75*), Fig. 3C]. The analysis shows that, when incorporating the data generated by the phage display experiment, both deep learning– and distance-based methods achieved accurate predictions. An alternative approach, which does not involve any computational modeling, consists of simply checking which of the 30 tested CDR3β has an exact match in the TCRs observed in the phage display experiments. We obtained an AUC of 0.58 with this approach (Fig. 3D). We next evaluated whether deep learning– and distance-based methods that do not include the phage display data generated in this study could identify the validated NY-ESO-1–specific TCRs. Both the pretrained NetTCR2.2 (*68*) and epiTCR (*74*) achieved only limited performance in this case with AUCs of 0.62 and 0.52 (Fig. 3E). We also assessed the sequence similarity of the 30 TCRs with the reference CDR3β (CASSYVGNTGELFF) using TCRbase (*57*) and tcrdist3 (*75*). Some validated CDR3β sequences have high sequence similarity to the reference CDR3β, while others have lower sequence similarity. We obtained an AUC of 0.79 for TCRbase and of 0.68 for tcrdist3 (Fig. 3F).

Overall, this benchmark shows that combining phage display data with computational predictors of TCR-epitope interactions represents a promising strategy for identifying TCRs binding to NY-ESO-1 within a pool of potential candidates.

### TCRs identified by MixTCRpred display activity toward NY-ESO-1 and no detectable cross-reactivity

To investigate the functionality of the TCRs predicted by MixTCRpred to bind to NY-ESO-1, we conducted multiple cellular activation assays. To this end, we selected three TCRs: one observed in the phage display data (CDR3β: CASS**NL**G**GL**GELFF) and two high-scoring predictions (CDR3β: CASSYVGN**N**GELFF, CASSYVG**HR**GELFF; see Table 2), as well as the template 1G4 (CDR3β: CASSYVGNTGELFF). All these TCRs were among the top MixTCRpred predictions and were validated as NY-ESO-1 binders on the 1G4 template.

**Fig. 4.**
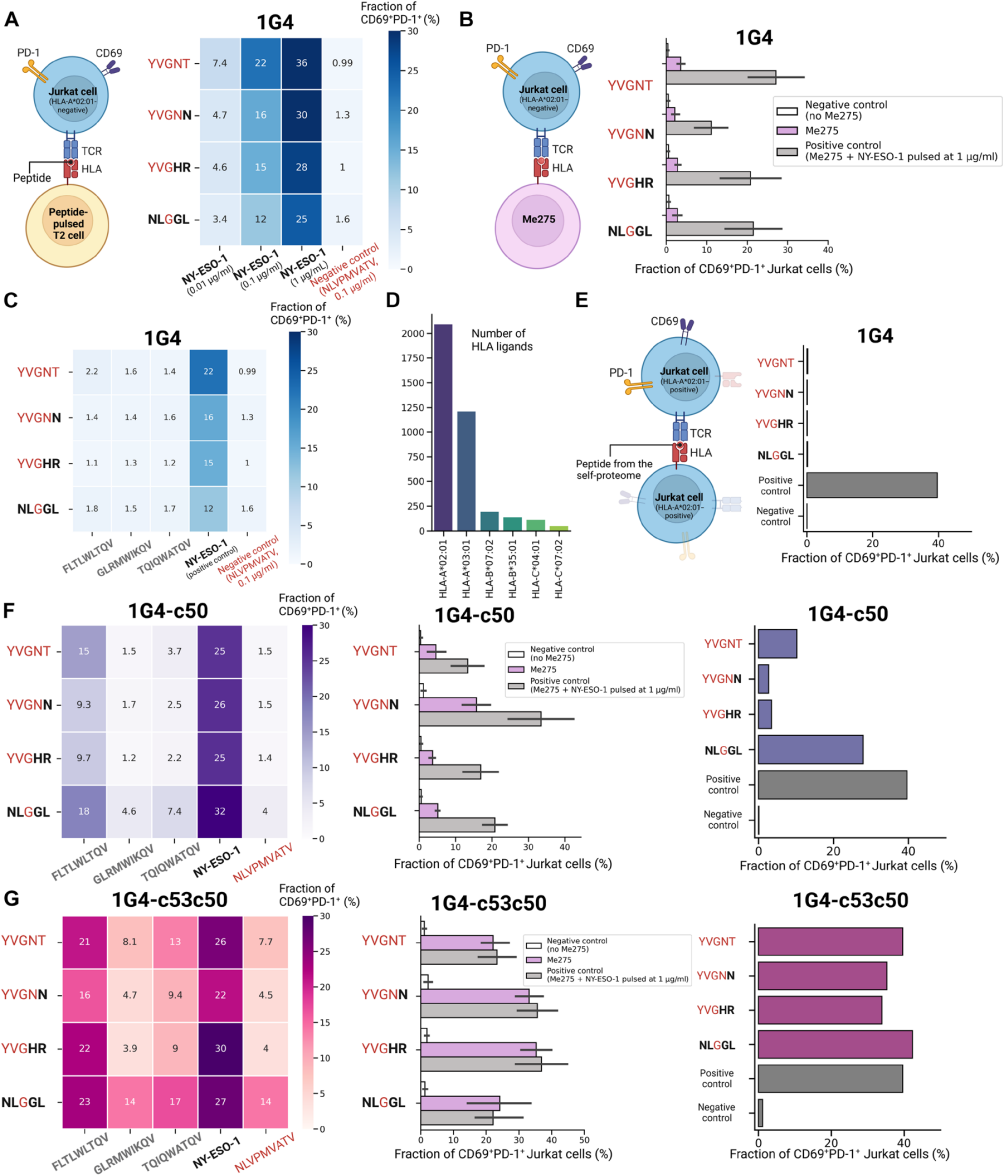
TCRs identified by MixTCRpred display activity toward NY-ESO-1 and no detectable cross-reactivity. (**A**) Heatmap showing the fraction of CD69^+^PD-1^+^ Jurkat cells encoding four TCRs on the 1G4 template after coculture with T2 cells pulsed with NY-ESO-1 at different concentrations. The CMV-derived epitope NLVPMVATV served as negative control. Mean values from three independent experiments are shown. The core region of the CDR3β loop is shown in the heatmap. (**B**) Fraction of CD69^+^PD-1^+^ Jurkat cells activated by coculture with HLA-A*02:01–positive Me275 melanoma cells expressing NY-ESO-1. Me275 pulsed with NY-ESO-1 at 1 μg/ml were used as positive controls; assays without Me275 served as negative controls. Mean values from two independent experiments in duplicate are shown. (**C**) Heatmap showing the fraction of CD69^+^PD-1^+^ Jurkat cells after coculture with T2 cells pulsed with three peptides from the self-proteome at peptide concentration of 0.1 μg/ml. NY-ESO-1 served as positive control and NLVPMVATV as negative control. Data from three independent experiments; the mean values are shown. (**D**) Number of HLA ligands identified by MS in HLA-A*02:01–transduced Jurkat cells and predicted to bind to the transduced or the endogenous HLAs. (**E**) Fraction of CD69^+^PD-1^+^ Jurkat cells activated by coculturing with HLA-A*02:01–transduced Jurkat cells presenting peptides derived from the self-proteome. Jurkat cells without any TCR expression were used as negative controls, while Jurkat cells expressing the affinity-enhanced TCR 1G4-c53c50 were used as positive controls. Mean values from three independent experiments in duplicate are shown. (**F** and **G**) Fraction of CD69^+^PD-1^+^ Jurkat cells expressing the four TCRs on the affinity-enhanced 1G4-c50 (F) and 1G4-c53c50 (G) templates under three conditions: stimulation with the indicated peptide at 0.1 μg/ml, coculture with Me275 cells, and stimulated by peptides derived from the self-proteome. Created in BioRender. G. Croce (2025), https://BioRender.com/z88e433.

We synthesized DNA encoding for these TCRs and transduced them into Jurkat cells that do not express the HLA-A*02:01 complex (HLA-A*02:01–negative J76 CD8αβ cell line). Jurkat cells were cocultured overnight with HLA-A*02:01–positive T2 cells pulsed with the NY-ESO-1 peptide. Activation markers CD69 and programmed cell death protein 1 (PD-1) were used to identify peptide-activated T cells. This experiment showed that Jurkat cells expressing any of the four TCRs were specifically activated by the NY-ESO-1 epitope at multiple peptide concentrations (0.01, 0.1, and 1 μg/ml) (Fig. 4A). On the contrary, these cells were minimally activated (close to zero fraction of CD69^+^PD-1^+^ T cells) by the cytomegalovirus (CMV)–derived epitope NLVPMVATV at peptide concentration of 0.1 μg/ml, which was used as a negative control.

Next, we evaluated whether the predicted TCRs could be activated in the same level as the 1G4 template by a melanoma cell line (Me275), which is HLA-A*02:01–positive and capable of presenting the NY-ESO-1 epitope from its endogenous proteome (*76*). As controls, we included Jurkat cells without Me275 cells (negative control) and Jurkat cells cocultured with Me275 cells pulsed with a high concentration (1 μg/ml) of exogenous NY-ESO-1 peptide (positive control). These experiments demonstrated that the predicted TCRs exhibited activation levels similar to those observed for the 1G4 (Fig. 4B).

To assess putative cross-reactivity, we investigated whether our TCR-transduced Jurkat cells could be activated by self-peptides displaying similarity to NY-ESO-1. We first selected three peptides from the human proteome (**FLTLWLTQV** from the proliferation marker protein Ki-67 - KI67_HUMAN; **GLRMWIKQV** from the lysophospholipase-like protein 1 - LYPL1_HUMAN; and **TQIQWATQV** from the conserved oligomeric Golgi complex subunit 7 - COG7_HUMAN), which were predicted to be presented by HLA-A*02:01 and have high sequence similarity with the NY-ESO-1 epitope (SLLMWITQC, see Methods). Two of them (FLTLWLTQV and TQIQWATQV) were also reported in an earlier study investigating the binding properties of the affinity-enhanced TCR NY-ESOc259 (*37*). Moreover, it has been reported that the FLTLWLTQV peptide, derived from a protein ubiquitously expressed in dividing cells, is not always efficiently processed and presented on the HLA complex (*77*). The peptides were pulsed on HLA-A*02:01–positive T2 cells and incubated overnight with Jurkat cells transduced individually with the three predicted TCRs (i.e., CDR3β loops CASSYVGN**N**GELFF, CASSYVG**HR**GELFF, and CASS**NL**G**GL**GELFF on the 1G4 template). We observed specific activation by the NY-ESO-1 epitope and close to zero activation by the other three peptides. The residual cross-reactivity was even lower than for the template TCR 1G4 with the reference CDR3β (CASSYVGNTGELFF) (Fig. 4C). These results were confirmed at various peptide concentrations (figs. S9 and S10).

To broaden our cross-reactivity investigation beyond the few selected peptides, we performed a functional assay to evaluate T cell cross-reactivity toward peptides from the self-proteome. Jurkat cells were dually transduced for both TCRs and HLA-A*02:01 and maintained under steady-state culture conditions for 3 to 6 days (HLA-A*02:01–positive J76 CD8αβ cell line). Such cells spontaneously present epitopes derived from a multitude of endogenously expressed proteins. Using mass spectrometry (MS)–based immunopeptidomics (see Methods), we determined that this cell line can present over 4000 different self-peptides, including more than 2000, which could confidently be assigned to the transduced HLA-A*02:01 molecule (Fig. 4D and data S3). Jurkat cells without any TCR expression (no transduction) were used as negative controls while cells expressing the high-affinity TCR 1G4-c53c50 were used as positive control (see Methods). The three predicted TCRs mirrored the template 1G4 TCR in triggering minimal T cell activation in this assay (close to zero fraction of CD69^+^PD-1^+^ T cells), suggesting a strong retention of specificity for the cognate NY-ESO-1 peptide (Fig. 4E).

We next repeated these experiments with the three predicted TCRs, and the one with the reference CDR3β, this time based on the affinity-enhanced templates 1G4-c50 and 1G4-c53c50. We observed that TCR-transduced Jurkat cells were functionally activated by the NY-ESO-1 peptide but displayed cross-reactivity with the FLTLWLTQV and, to a lesser extent, with the GLRMWIKQV and TQIQWATQV peptides (Fig. 4, F and G, and figs. S9 and S10). These TCR-Jurkat cells were activated during coculture with Me275 cancer cells, but they demonstrated extensive cross-reactivity with peptides derived from the Jurkat self-proteome (Fig. 4, F and G).

Overall, this analysis reveals that TCRs predicted by MixTCRpred exhibit activity toward NY-ESO-1 and no detectable cross-reactivity. Conversely, cross-reactivity with both specific peptides and the peptidome was detected when using the affinity-enhanced 1G4-c50 and 1G4-c53c50 TCR templates.

### Structural analyses reveal the molecular basis of the CDR3β binding motifs

**Fig. 5.**
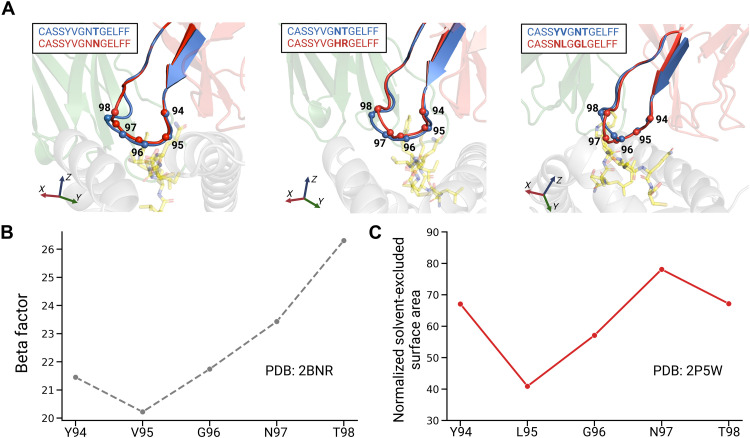
Structural analyses reveal the molecular basis of the CDR3β binding motifs. (**A**) Predicted binding modes of three CDR3β loops (CASSYVGNNGELFF, CASSYVGHRGELFF, and CASSNLGGLGELFF, in red), overlapped with the reference CDR3β (CASSYVGNTGELFF, in blue). The molecular modeling was performed using the crystal structure of the 1G4 template (PDB: 2BNR). The α-carbons of the core region of the CDR3β loops are represented as spheres. (**B**) Beta factor of the core region of the CDR3β loop (PDB: 2BNR). (**C**) Normalized solvent-excluded surface area (SESA) of amino acid side chains in the core region of CDR3β loop in the 1G4-c53c50 template (PDB: 2P5W, residue numbering based on PDB: 2BNR). Created in BioRender. G. Croce (2025), https://BioRender.com/m22r428.

To gain molecular insights into the binding motifs of NY-ESO-1–specific TCRs, we first attempted to model the three TCRs functionally validated in Fig. 4A (CDR3β: CASSYVGN**N**GELFF, CASSYVG**HR**GELFF, and CASS**NL**G**GL**GELFF) using as template the crystal structure of 1G4 [Protein Data Bank (PDB): 2BNR; (*78*)]. Overall, the CDR3β loops are predicted to maintain a conformation similar to that of 1G4 (CDR3β: CASSYVGNTGELFF). Even in the case of four point mutations (**NL**G**GL** versus YVGNT), the mutations Y94N and V95L in the first two residues did not noticeably alter the positions of the α-carbons with respect to the reference CDR3β. Slightly more pronounced structural rearrangements are predicted to occur toward the end of the core regions of the CDR3β loop (Fig. 5A), which is consistent with the lower amino acid specificity at these positions in the motifs of Fig. 1F. We then analyzed the beta factors of Cα across the CDR3β loop (Fig. 5B). Higher beta factors were observed for the last two amino acids, which is consistent with the lower specificity and higher flexibility observed in the motif and structural models of CDR3β loops with a core region of length 5.

We next explored potential mechanisms for the longer CDR3β loops observed in the CDR3β binding motifs based on affinity-enhanced templates (Fig. 1F) and in some of the TCRs validated on these templates (Fig. 2D). To determine where the CDR3β loops may accommodate the additional residues, we analyzed the solvent accessibility of amino acid side chains in the core region of the CDR3β loop for the 1G4-c53c50 template (PDB: 2P5W). In this crystal structure, Leu is found at the second position (L95) instead of Val, which is compatible with the sequence motifs we identified in the phage display experiments (Fig. 1F). We observed that amino acids located in the C-terminal region of the core of the CDR3β loop (positions 97 and 98) are more solvent-exposed (Fig. 5C). This suggests that the loop extension occurs toward the end of the core region of the CDR3β loop. This is consistent with the longer motifs for the affinity-enhanced templates that were obtained in the phage display experiments (Fig. 1F). Overall, our structural analyses provide a molecular interpretation for the high specificity and conservation across lengths of the N-terminal part of the binding motifs in Fig. 1F and the lower specificity at the C-terminal part.

## DISCUSSION

Phage display provides a powerful framework to screen very large libraries of TCRs with randomized amino acids at specific positions against clinically relevant epitopes. In this work, we demonstrate that phage display can be used in combination with machine learning predictors of TCR-epitope interactions to identify epitope-specific TCRs directly from native TCR repertoires. Our MixTCRpred computational model could, therefore, facilitate and accelerate the identification of multiple TCRs for adoptive transfer in cancer immunotherapy. Such diversity in TCRs may offer therapeutic advantages compared to using only one high-affinity TCR (*79*, *80*).

Our phage display approach relies on having at least one TCR specific for the epitope under investigation, referred to as the template TCR, from which a library with randomly mutated CDR3β loops can be designed and selected against a specific epitope. Analysis of epitope-specific TCRs in VDJdb shows that this is the case for more than 1000 epitopes. The phage display approach is particularly valuable for cancer epitopes for which cognate TCRs are typically challenging to experimentally identify in primary T cells from patients or healthy donors. These include the cancer-testis antigen NY-ESO-1_157–165_.

Sequencing data from high-throughput phage display screening can contain a large number of putative contaminants. Our results show that motif deconvolution algorithms like MoDec (*72*) can be effectively used to unravel binding motifs even in the presence of a substantial fraction of unspecific TCRs. The high similarity of the motifs observed in all three templates as well as our multiple experimental validations demonstrate that motifs identified by motif deconvolution in this work represent bona fide CDR3β binding motifs of TCRs specific for the NY-ESO-1 epitope.

Earlier studies (*42*, *43*) used iterative selection and amplification of phage libraries to identify TCRs with enhanced affinity for different epitopes by exploring amino acid substitutions at multiple positions across the TCR-epitope interaction interface (i.e., not restricted to the variable region of the CDR3 loops). As a result, many of the V segments of these TCRs deviate from those of native TCRs and have not undergone positive and negative selection in the thymus. This increases the risk of cross-reactivity with self-peptides, as demonstrated in this work for the affinity-enhanced 1G4-c50 and 1G4-c53c50 templates, potentially leading to toxicity in clinical applications (*49*). Native TCRs from TCR repertoires represent promising candidates for the development of T cell–based immunotherapies (*79*, *81*), having a lower risk of cross-reactivity and, thus, a reduced risk of adverse reactions compared to affinity-enhanced TCRs. Our proposed strategy, consisting of designing phage display libraries specific for the most variable region of the CDR3β loop and using the data for training TCR-epitope interaction prediction tools to interrogate TCR repertoires, enabled us to rapidly identify multiple candidates within tens of thousands of TCR sequences. Our results confirmed that the predicted TCRs exhibit functional activation similar to that of 1G4. Although we cannot rule out potential cross-reactivity in vivo, including with peptides presented across various human tissues by diverse HLAs not expressed in our Jurkat cells or due to the inherent complexity of HLA presentation mechanism, our experiments allowed us to exclude cross-reactivity with a considerable portion of the self-peptidome.

Our study shows that high accuracy in predicting NY-ESO-1–specific TCRs can be achieved by combining our high-throughput experimental phage display pipeline together with a customized machine learning model. Other approaches that either do not use the phage display data or do not have a dedicated machine learning model achieved lower accuracies, as demonstrated in our internal benchmark. For this benchmark, we used experimentally tested TCRs with a broad range of MixTCRpred scores, thereby mitigating biases toward the high-score predictions. We cannot exclude that some high-scoring TCRs with other tools and with CDR3β sequences incompatible with the phage-derived motifs may still bind but were not included in our test set. However, the depth of the phage display libraries suggests that these cases should be rare.

The binding motifs derived from the phage display experiments with different templates displayed high similarity. This observation has several important consequences. First, it shows that affinity-enhanced templates are not necessary for the pipeline proposed in this work. As such, many native TCRs binding to clinically relevant epitopes in public databases could be used as templates for designing phage display libraries similar to the one built in this work. Second, it shows that affinity-enhanced templates, which displayed extensive cross-reactivity and, therefore, would not be suitable for any clinical application, are compatible with the training of predictors and do not lead to artifacts when scoring TCRs from TCR repertoires. This may be useful when working with clinically relevant epitopes for which only very low affinity TCRs are known and which could not be used directly in phage display.

In our phage display libraries, we diversified the core region of the template CDR3β loop across different lengths, keeping the V and J genes as well as the CDR3α loop unmodified. As a result, the MixTCRpred model trained on such data is only applicable to make predictions for TCRβs with these specific V and J genes. When testing other V genes (including some with high sequence similarity to the TRBV6-5*01 template), we did not observe any binding, which explains why our predictions could not be expanded to other V segments. While this represents a limitation of the current study, we anticipate that the proposed combined experimental and computational framework will be useful for designing and screening phage display libraries built with other V and J genes and/or including diversification in the CDR3α loop.

In summary, our work presents an integrated experimental and computational pipeline to identify TCRs recognizing clinically relevant epitopes. In particular, we demonstrate that combining phage display with machine learning enabled us to train TCR-epitope interaction predictors for a clinically relevant epitope for which only few TCRs had been identified by other means. We anticipate that this work will pave the way for designing larger TCR libraries encoded in phage or other organisms (*82*, *83*) to expand the epitope coverage of TCR-epitope interaction predictors and facilitate the identification of epitope-specific TCRs directly from TCR repertoires of patients with cancer or healthy donors.

## METHODS

### Randomized CDR3β library

Randomized CDR3β libraries of DNA of differing lengths were constructed on the 1G4 single-chain TCR (scTCR) scaffold. Briefly, 1G4 TCR domains were codon-optimized for expression in *Escherichia coli* and cloned as a three-domain Vα-VβCβ single-chain ORF fused N-terminally to the M13/f1 gIII major coat protein in a pUC19-based phagemid vector (pCHV101). In addition to parental 1G4, which has an affinity for its cognate antigen (NY-ESO-1_157–165_) of ~10 μM (*38*–*40*), we also included two variants containing CDR2 “anchoring” mutations that have been shown to increase the affinity of the TCR to ~0.1 and ~0.001 μM, respectively, through improved contacts with the common HLA-A*02:01 helices that flank the peptide groove (*43*). To circumvent the reported instability conferred by the Cys at 3′ of the NY-ESO-1 peptide, we conducted all phage library selection using the more stable A0201_SLLMWITQ**V** pMHC complex (*78*). Using these scTCR DNA templates, the core region of the 1G4 CDR3β (YVGNT) was diversified in a two-step PCR using standard procedures. The initial PCRs used a common forward primer and discrete reverse primers incorporating tails comprising different lengths of trimer-defined (TRIM) codons (Ella Biotech GmbH, Martinsried, Germany). We interrogated the publicly accessible VDJdb database (*27*) comprising some 40,000 human TCRs targeting ~1000 distinct MHC antigens. From these data, a single–amino acid codon mixture was devised to approximate the composition of natural CDR3β loop cores (table S1). These individual length-variant PCR products were then used as templates in a second PCR reaction to introduce an Xho I restriction site downstream of the diversified CDR3 (fig. S1 and table S2).

### Phage library

The diversified library PCR fragments were double digested with Asc I (upstream of CDR3β) and Xho I and ligated into the similarly digested pCHV101 template vectors. Ligated products were purified and used to electroporate electrocompetent *E. coli* TG1 cells (Lucigen), which were then directly plated onto large 2x yeast extract tryptone medium (2TY)-agar plates supplemented with ampicillin (100 μg/ml)/2% glucose and incubated at 30°C overnight. The following day, the bacterial libraries (all of size > 10^8^ clones) were harvested from plates and stored as concentrated glycerol stocks at −80°C. Filamentous phage libraries were rescued using M13 helper phage (Life Technologies), concentrated and purified according to the standard procedures, and stored in aliquots at −80°C. The physical size of the constructed libraries actually realized (number of bacterial clones on plates) were 2.7 × 10^8^, 2.6 × 10^8^, and 6.1 × 10^8^ for 5-mers, 7-mers, and 9-mers, respectively. To assess diversity, the libraries were sequenced with 2 × 250–base pair (bp) paired-end next-generation sequencing (NGS) using an in-house Illumina MiSeq platform, requesting one million reads per library (data S1). Clones (37.5, 69, and 49.8%) in each library were determined to be functional intact clone opening reading frames identical to the parental TCR.

### Phage panning

Phage panning of the libraries was performed according to standard procedures against monomeric biotinylated HLA-A*02:01,NY-ESO_157–165_ (heteroclitic peptide variant: SLLMWITQV) immobilized on M-280 streptavidin magnetic beads (Thermo Fisher Scientific), using an irrelevant bio-HLA-A*0201 peptide complex for deselection of nonspecific binders. One round of panning was conducted, incorporating different stringencies controlled by varying the number of wash cycles. The enrichment of specific CDR3β sequences was assessed by performing 2 × 250-bp paired-end NGS sequencing on the extracted and amplified output DNA using an in-house Illumina MiSeq platform and requesting one million reads in each sample.

### Phage display data processing

The TCR-sequencing data obtained from the Illumina MiSeq platform were processed with the MiXCR v3.0.13 with standard parameters (mixcr analyze amplicon --species hs starting-material dna --5-end v-primers --3-end j-primers --receptor-type TRB) (*84*). To ensure high-quality data for the phage display generated data, we removed all the TCR sequences occurring only once, which are likely due to sequencing errors. We also removed the “YVGNT” sequences from the phage display generated data, which could reflect template TCRs, which failed randomization. Duplicated sequences were removed, retaining only unique TCR clonotypes. The unsupervised motif deconvolution tool MoDec was used to further process the TCR sequences (*72*). We set the number of motifs to 1 (*k* = 1) and background frequencies based on the amino acid distribution in the input phage libraries. TCR sequences belonging to the flat motif or with motifs lacking positions with information content higher than 1 were considered as putative contaminants and removed (fig. S2 and data S2).

### MixTCRpred—Model architecture and data

We developed a MixTCRpred model customized for CDR3β sequence data used in this study. MixTCRpred is a deep learning model for TCR-epitope interaction prediction. MixTCRpred architecture consists of an embedding and positional encoding layer, followed by a transformer encoder, and a fully connected network for classification. To train the MixTCRpred model, we used as positives the phage display output sequences from the 1G4, 1G4-c50, and 1G4-c53c50 templates separately (598 positives for the 1G4 templates, 9889 for 1G4-c50, and 26,308 for 1G4-c53c50). As negatives (CDR3β nonbinding to NY-ESO-1), we used sequences from the phage input libraries that were not present in the output libraries. Additionally, we trained a model (A0201_NY-ESO-1-CDR3b) using the combined datasets and removing duplicate sequences, resulting in a total of 29,688 positives. We collected a large number of TCRβ sequences from TCR repertoires downloaded from iReceptor (*73*) with *TRBV6-5* and *TRBJ2-2* genes and filtered out sequences with N- and C-terminal parts different from that of the reference CDR3β (respectively, CASS and GELFF) or containing non-standard amino acids. In total, we retrieved 29,867 TCRβ sequences. Similarly, we downloaded from iReceptor (*73*), a large database of 10 million TCRβ with diverse V and J genes.

### TCR cloning and multimer staining

Thirty CDR3β with high MixTCRpred scores were selected for pMHC multimer staining experiments (Table 2). CDR3β sequences were based on the three template TCRs (1G4, 1G4-c50, or 1G4-c53c50; Table 1) and tested for binding versus the NY-ESO-1 epitope (HLA-A*02:01,SLLMWITQC) (Fig. 2D). TCRα/β pairs were cloned into Jurkat cells J1601 (TCR/CD3 stably transduced with human CD8α/β and TCRα/β CRISPR-KO) (*85*, *86*). Codon-optimized DNA sequences coding for paired α and β chains were synthesized at GeneArt (Thermo Fisher Scientific) or elesis Bio DNA. The DNA fragments served as template for in vitro transcription and polyadenylation of RNA molecules as per the manufacturer’s instructions (Thermo Fisher Scientific), followed by co-transfection into recipient T cells. Jurkat cells were electroporated using the Neon electroporation system (Thermo Fisher Scientific) with the following parameters: 1325 V, 10 ms, and three pulses. After overnight incubation, electroporated Jurkat cells were interrogated by pMHC-multimer staining with the following surface panel: anti-hCD3 allophycocyanin (APC) Fire 50 (SK7; BioLegend, catalog no. 641415; 0.4 μl in 50 μl); anti-hCD8 fluorescein isothiocyanate (FITC; SK-1; BioLegend, catalog no. 344704; 0.15 μl in 50 μl); anti-hCD4 phycoerythrin (PE)-CF594 (RPA-T4; BD Biosciences, catalog no. 562281; 0.4 μl in 50 μl); anti-mouse TCRβ-constant APC (H57-597; Thermo Fisher Scientific, catalog no. 17-5961-81; 0.6 μl in 50 μl); pMHC-multimer-PE (HLA-A*02:01 with the SLLMWITQC peptide; in-house synthesized, 1 μl in 50 μl); and viability dye Aqua (L34966; Thermo Fisher Scientific; 0.15 μl in 50 μl of staining mix in PBS). The peptides and pMHC multimers were produced by the Peptide and Tetramer Core Facility of the Department of Oncology, UNIL-CHUV, Lausanne. Samples were acquired by flow cytometry, and fluorescence-activated cell sorting data were analyzed with FlowJo 10.8.1 (TreeStar).

### Benchmarking MixTCRpred trained on phage display data with other approaches

We used the 30 experimentally tested TCRs with the 1G4 template (6 positives and 24 negatives) to benchmark our strategy with the following approaches:

1) TCRbase 1.0 (web server: https://services.healthtech.dtu.dk/services/TCRbase-1.0/). The 1G4 template sequence (Table 1) was used as the training database, and the list of the 30 tested TCRs was uploaded on the web server to compute the sequence similarity with the reference CDR3β (CASS**YVGNT**GELFF).

2) Tcrdist3 (GitHub page: https://github.com/kmayerb/tcrdist3). The function compute_distances from the tcrdist package was used to compute the sequence similarity of the 30 tested TCRs with the reference CDR3β (CASS**YVGNT**GELFF).

3) NetTCR2.2 (web server: https://services.healthtech.dtu.dk/services/NetTCR-2.2/). The web server was used to compute the likelihood of interaction between the NY-ESO-1 peptide and the 30 tested TCRs.

4) epiTCR (GitHub page: https://github.com/ddiem-ri-4D/epiTCR). The pretrained model models/rdforestWithoutMHCModel.pickle was used to compute the interaction score between the NY-ESO-1 peptide and the 30 tested TCRs.

5) pMTNet (web server: https://dbai.biohpc.swmed.edu/pmtnet/). The 30 tested TCRs sequences were uploaded on the web server to compute the likelihood of interaction with the NY-ESO-1 peptide.

### Culture of cell lines and generation of TCR-transduced J76 CD8αβ T cells

TCR knockout HLA-A*02:01^neg^/J76 CD8αβ cells (provided by I. Edes and W. Uckert, Max-Delbrück-Center, Berlin, Germany), TCR knockout HLA-A*02:01^pos^/J76 CD8αβ cells (*54*), and HLA-A*02:01^pos^ transporter associated with antigen processing (TAP)–deficient T2 cells [American Type Culture Collection (ATCC), CRL-1992] were cultured at 37°C and 5% CO_2_ in RPMI 1640 supplemented with 10% fetal calf serum, 10 mM Hepes, penicillin (100 U/ml), streptomycin (100 μg/ml), 1× nonessential amino acids, and 1 mM sodium pyruvate.

The full-length, codon-optimized AV23.1 and BV13.1 chain sequences of NY-ESO-1_157–165_–specific 1G4, 1G4-c50, and 1G4-c53c50 TCRs, separated by an IRES module, were synthetized by GeneScript and cloned into the MCS Bam HI/Xho I of self-inactivating gamma-retroviral vector (SFG retroviral vector). Additional amino acid substitutions within the CDR3β loops of 1G4, 1G4-c50, and 1G4-c53c50 TCRs were generated by introducing short 81-bp mutagenic single-stranded DNA fragments using the NEBuilder HiFi DNA Assembly protocol (BioLegend) according to the manufacturer’s instruction. Constructs were transformed into XL10-Gold ultracompetent bacteria (Agilent), and full-length TCRaβ sequences were confirmed by DNA sequencing.

Retroviral vectors were produced by transient transfection of 293 T cells in 100 μl of Dulbecco’s modified Eagle’s medium supplemented with 3% GeneJuice transfection reagent (Sigma-Aldrich) with the vector of interest (SFG.TCR AV23.1-IRES-TCR BV13.1) and the PegPam3 (gag-pol) and RDF (env) plasmids. Supernatant of retroviral-transfected 293 T cells was used to transduce HLA-A*02:01^neg^ or HLA-A*02:01^pos^ J76 CD8αβ cells using RetroNectin (Takara)–coated plates. TCR-positive HLA-A*02:01^neg^ or HLA-A*02:01^pos^ J76 CD8αβ cells were sorted to purity by flow cytometry (FACSAria II and III, BD Biosciences) using PE-labeled A2/NY-ESO-1_157-165–specific multimers (Peptide & Tetramer Core Facility, UNIL-CHUV, Lausanne) and anti-Vβ13.1 APC antibodies (BioLegend).

### NY-ESO-1 specificity and cross-reactivity functional assays

HLA-A*02:01^pos^ TAP–deficient T2 cells were loaded with three peptides (FLTLWLTQV, GLRMWIKQV, and TQIQWATQV) from the self-proteome, with NY-ESO-1 (SLLMWITQA) or the CMV/pp65 (NLVPMVATV) peptide at 0.01, 0.1, or 1 μg/ml at 37°C for 1 hour. We used the heteroclitic SLLMWITQA peptide instead of SLLMWITQC to avoid disulfide bridge formation, improving loading onto HLA complexes and T cell responses (*35*, *87*). Peptide-loaded T2 targets were cocultured with TCR-transduced HLA-A*02:01^neg^ J76 CD8αβ cells at a 3:1 ratio (1.5 × 10^5^ T2 and 0.5 × 10^5^ J76) during 16 hours in U-bottom 96-well plates. Cocultures using unloaded “empty” T2 targets or parental HLA-A*02:01^neg^ J76 cells without any TCR expression (no transduction) were used as additional negative controls, while TCR-transduced J76 cells stimulated with phorbol 12-myristate 13-acetate (500 ng/ml)/ionomycin (250 ng/ml) were used as additional positive control (figs. S9 and S10). In parallel, HLA-A*02:01^pos^ Me275 cells naturally expressing NY-ESO-1_157–165_ were cocultured with TCR-transduced HLA-A*02:01^neg^ J76 CD8αβ cells at a 3:1 ratio (1.5 × 10^5^ Me275 and 0.5 × 10^5^ J76) during 16 hours in U-bottom 96-well plates. HLA-A*02:01^neg^ J76 cells cultured alone were used as negative controls. Me275 cells loaded with NY-ESO-1 (1 μg/ml; SLLMWITQA) peptide were used as positive controls. To assess T cell activation, cells were stained at room temperature with anti-CD69 peridinin-chlorophyll-protein complex (PerCP)-eF710 (Invitrogen), anti–PD-1 PE (BioLegend), and anti-Vβ13.1 APC (BioLegend) antibodies for 20 min. For the peptide-pulsed T2 assays, anti-CD20 FITC (BioLegend) was added to gate out the CD20^+^ antigen presenting cells. 4′,6-Diamidino-2-phenylindole was used as a dead cell marker. Samples were acquired on a CytoFLEX (Beckman Coulter) flow cytometer, and data were analyzed by FlowJo software (TreeStar, v.10.8.1). The gating strategy is illustrated in fig. S11.

### Self-proteome reactivity assay

Freshly TCR-transduced HLA-A*02:01^pos^ J76 CD8αβ cells (5 × 10^4^) were cultured during 3 to 6 days in U-bottom 96-well plate under steady-state culture conditions for testing the presentation of endogenous epitopes derived from the self-proteome. Parental HLA-A*02:01^pos^ J76 cells without any TCR expression (no transduction) were used as negative controls, while HLA-A*02:01^pos^ J76 cells expressing the high affinity 1G4-c53c50 TCR variant were used as positive control (Fig. 4). T cell activation was assessed by flow cytometry as described in the previous section.

### Selection of peptides showing high similarity to NY-ESO-1

The human proteome data, excluding isoforms, was downloaded from UniProt (www.uniprot.org/proteomes/UP000005640), from which we generated all possible 9-mers MixMHCpred (*85*) was then used to predict the binding affinity of these peptides to HLA-A*02:01. Only cases having %rank values below 1.5 were selected for further analysis. Next, we computed the sequence similarity between a peptide and the NY-ESO-1 epitope (SLLMWITQC) using the BLOSUM62 scoring matrix (*88*) from the biopython package (*89*). Among the top scoring cases, we selected three peptides (FLTLWLTQV, GLRMWIKQV, and TQIQWATQV) having specific residues (W5 and Q8), which are known to be critical for TCR binding. The FLTLWLTQV and TQIQWATQV peptides were also reported in an earlier study investigating the cross-reactivity of the affinity-enhanced TCR NY-ESOc259 (*37*).

### Purification of HLA bound peptides

HLA-I peptides of Jurkat cells (HLA-A*02:01^pos^ J76 CD8αβ cells) were immunopurified as previously described (*90*). Briefly, 10 million cells per biological replicate were lysed for 1 hour on ice and centrifugated for 30 min at 4°C in a tabletop centrifuge (Eppendorf) at 21,000*g*. The cleared lysates were loaded into wells of filter plates (Agilent) containing protein-A sepharose beads (Invitrogen) cross-linked with W6/32 antibodies purified from HB95 hybridoma cells supernatants (HB-95, ATCC). After several washes of varying concentrations of salts using the positive pressure manifold, HLA-I complexes were eluted using 1% trifluoroacetic acid (TFA; Sigma Aldrich) into preconditioned wells of Sep-Pak tC18 100 mg of Sorbent 96-well plates (Waters, ref. no: 186002321). After washing the C18 sorbents with 2 ml of 0.1% TFA, HLA-I peptides were eluted with 28% acetonitrile (ACN; Chemie Brunschwig) in 0.1% TFA, dried using vacuum centrifugation (Concentrator plus, Eppendorf), and stored at −20°C.

### Liquid chromatography and mass spectrometry

Immunopeptides were resuspended in 2% ACN and 0.1% formic acid. iRT peptides (Biognosis, Schlieren, Switzerland) were spiked into the samples (Biognosis) as recommended by the vendor and analyzed by liquid chromatography (LC)–tandem MS (MS/MS). The LC-MS system consisted of an Easy-nLC 1200 (Thermo Fisher Scientific, Bremen, Germany) coupled to Eclipse tribrid mass spectrometer (Thermo Fisher Scientific, San Jose, USA). HLA-bound peptides were eluted on a 450- to 500-mm analytical column (~8-μm tip, 75-μm inside diameter) packed with ReproSil-Pur C18 (1.9-μm particle size, 120-Å pore size, Dr. Maisch, GmbH) and separated at a flow rate of 250 nl/min as described (*90*).

For data-independent acquisition (DIA), DIA cycle of acquisition was performed on the Eclipse tribrid MS. The cycle of acquisitions consists of a full MS scan from 300 to 1650 mass/charge ratio (*m*/*z*; *R* = 120,000), ion accumulation time of 60 ms, normalized AGC of 250%, and 22 DIA MS/MS scans in the orbitrap. For each DIA MS/MS scan, a resolution of 30,000, a normalized AGC of 2000%, and a stepped normalized collision energy (*27*, *30*, *32*) were used. The maximum ion accumulation time was set to auto, the fixed first mass was set to 200 *m*/*z*, and the overlap between consecutive MS/MS scans was 1 *m*/*z*.

### Immunopeptidomics data analysis

A broad immunopeptidome spectral library was used to achieve a comprehensive analysis of the immunopeptidome (*91*). The library was imported to and analyzed by Spectronaut V19.2 (Biognosis, Schlieren, Switzerland). Briefly, DIA data analysis parameters were kept by default (BGS Factory setting) unless specified as follows. For peptide identification, precursor *Q*-value cutoff was set to 0.01, and, for experiment and run, protein *Q*-value cutoff was set to 1.00. For peptide quantification, cross-run normalization was unchecked, and minor grouping was set to peptide level (stripped sequence). For protein interference, workflow was set to automatic, and all-matching protein inference algorithm was used. Last, minor grouping (peptide level) was used for differential abundance analysis of peptides. The resulting analysis was then exported into peptide centric table. The list of peptides was analyzed using MixMHCpred2.2 (*85*) to identify peptides binding to the following HLA alleles: HLA-A*02:01, HLA-A*03:01, HLA-B*07:02, HLA-B*35:01, HLA-C*04:01, and HLA-C*07:02. Only peptides with a percentile rank lower than 0.5 were included in the final dataset.

### Structural analysis

Structural modeling of the TCR-pMHC complexes with modified CDR3β sequences was performed using the Modeller software (*92*) (version 10.2). The 2BNR crystal structure served as a template, with CDR3β sequences integrated into the TCR while maintaining the fixed coordinates of the pMHC and non-CDR3β TCR residues. Following this, the best five models were selected on the basis of the discrete optimized protein energy (DOPE) score evaluated for the TCR-pMHC interface encompassing CDR loops, the peptide, and MHC residues located within 6 Å from the peptide. Among the top five models, the one with the maximal number of hydrogen bonds and hydrophobic contacts was retained. The solvent accessibility of each CDR3β residue was determined as the relative solvent-excluded surface area (SESA) computed with the MSMS package of the UCSF Chimera software (*93*–*95*). The normalized SESA (nSESA) was calculated by normalizing the surface area of the residue in the TCR of interest by its surface area in a reference state. The latter was defined as the Gly-X-Gly tripeptides in which X is the residue type of interest (*96*). Thus, nSESA ranges from 0% for totally buried residues to 100% for residues exposed to the solvent to the same degree as in Gly-X-Gly. The beta factor of the core region of the CDR3β loop for the 1G4 template was obtained from the PDB entry 2BNR.
