# Supplementary material for: Phage display enables machine learning discovery of cancer antigen–specific TCRs
Source: Sci Adv. 2025 Jun 11;11(24):eads5589. doi: 10.1126/sciadv.ads5589 (PMC12225687; doi:10.1126/sciadv.ads5589)
Supplement: Supplementary file 1 — Figs. S1 to S11 Tables S1 to S6 Legends for data S1 to S3 [file sciadv.ads5589_sm.pdf]

Science Advances
AAAS

Supplementary Materials for

Phage display enables machine learning discovery of cancer antigen–specific TCRs

Giancarlo Croce *et al.*

Corresponding author: Steven M. Dunn, steven.dunn@chuv.ch; David Gfeller, david.gfeller@unil.ch

Sci. Adv. **11**, eads5589 (2025)
DOI: 10.1126/sciadv.ads5589

The PDF file includes:

Figs. S1 to S11
Tables S1 to S6
Legends for data S1 to S3

Other Supplementary Material for this manuscript includes the following:

Data S1 to S3

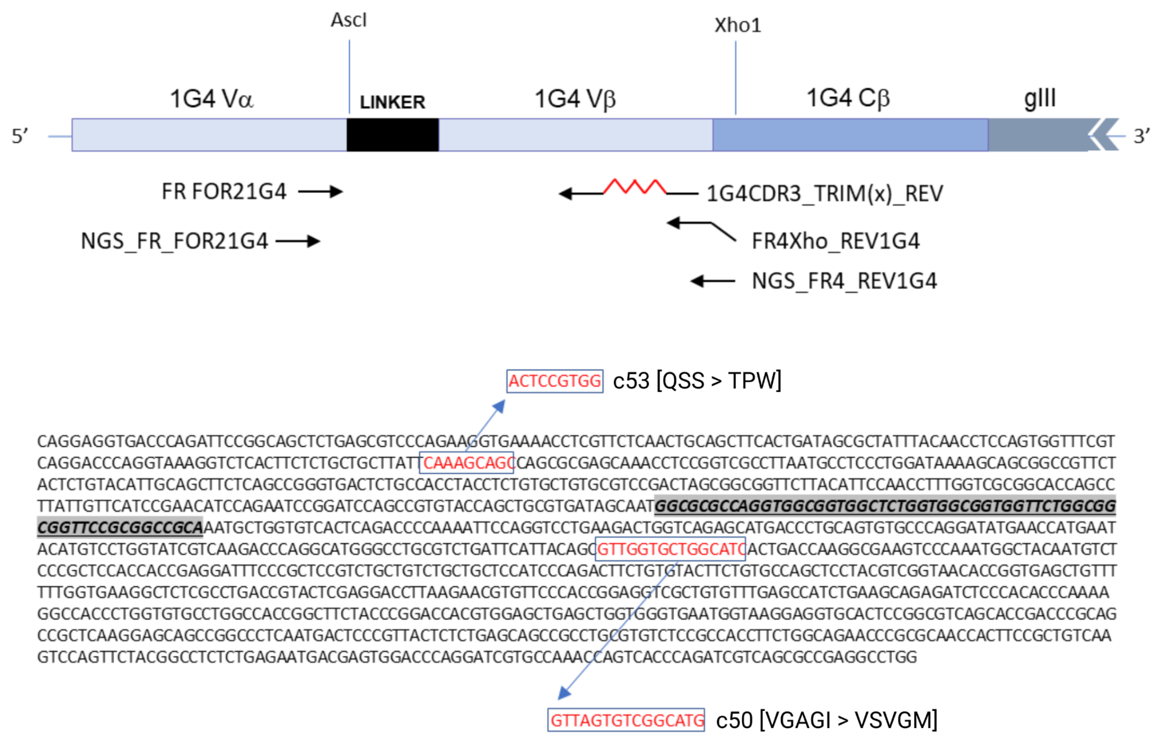

Fig. S1. Schematic of 1G4 CDR3β PCR strategy (upper), and codon-optimized DNA sequence and variants (lower).

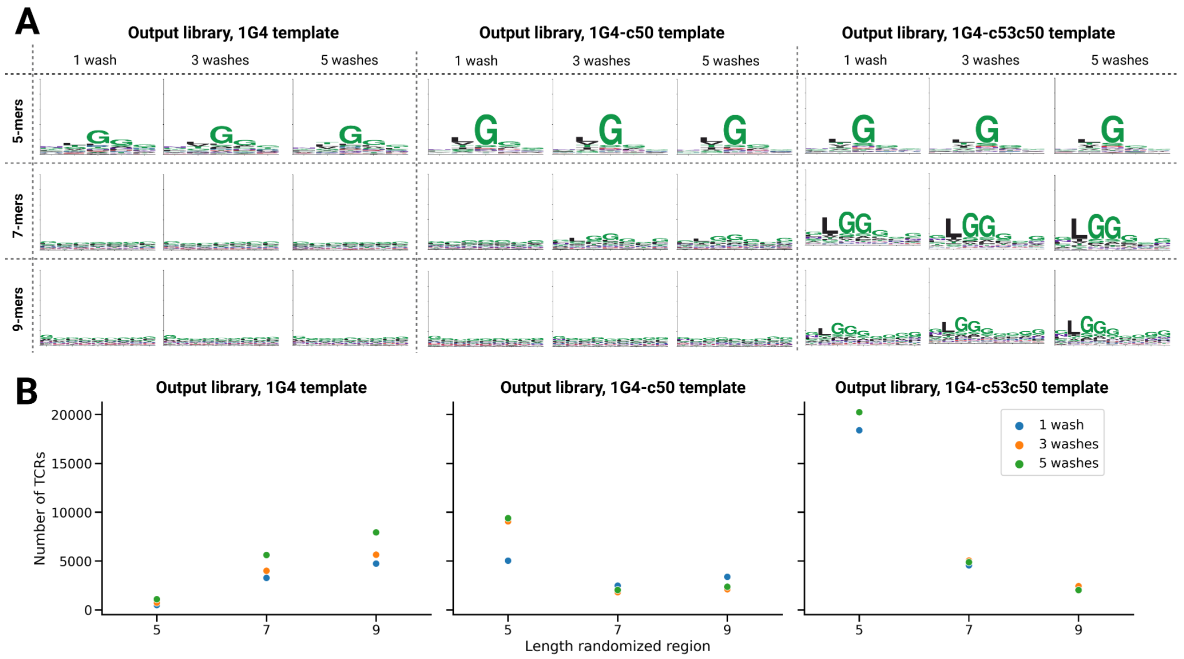

Fig. S2. Output libraries of the phage display screening. (A) Motifs of the raw TCR sequences resulting from the phage display screening with 1,3, and 5 washes. The data for each template TCR are shown separately. (B) Number of TCR sequences of different lengths resulting from the phage display screening with 1, 3 and 5 washes.

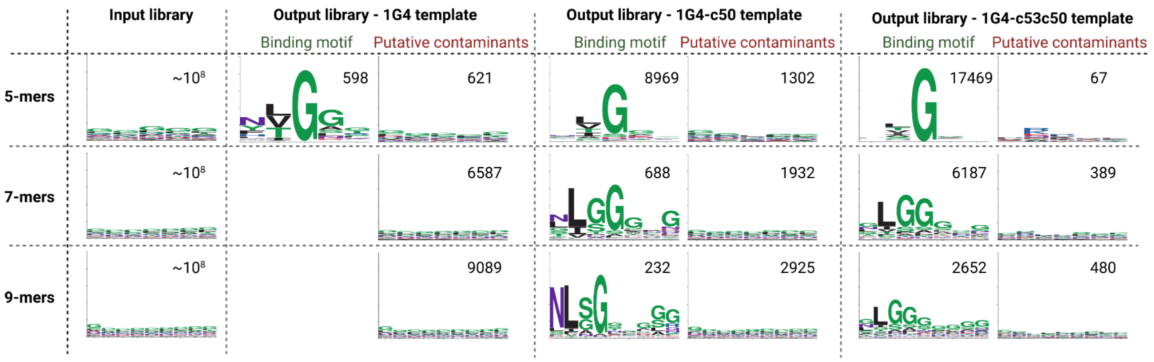

Fig. S3. Motifs of the input and output libraries of the phage display screening after filtering out putative contaminants with the motif-deconvolution algorithm MoDec (*72*). For each motif, the number of TCR sequences is also reported.

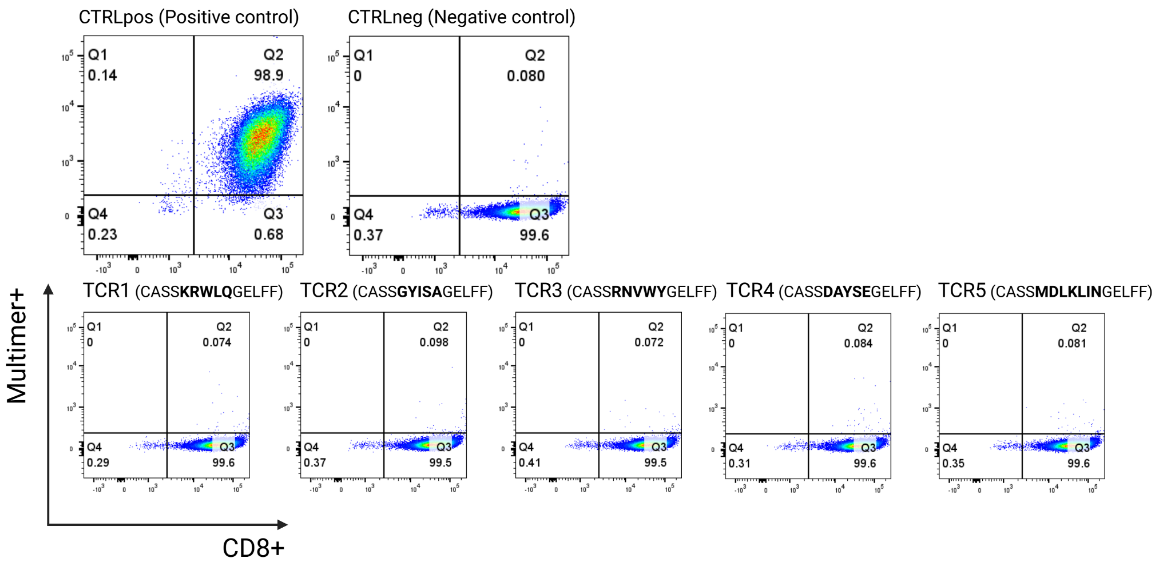

Fig. S4. FACS plots showing the results of multimer staining for five randomly selected TCRs predicted to be contaminants by MoDec. The corresponding CDR3β sequences are provided in parentheses (see also table S4). The results for the positive and negative controls are also shown.

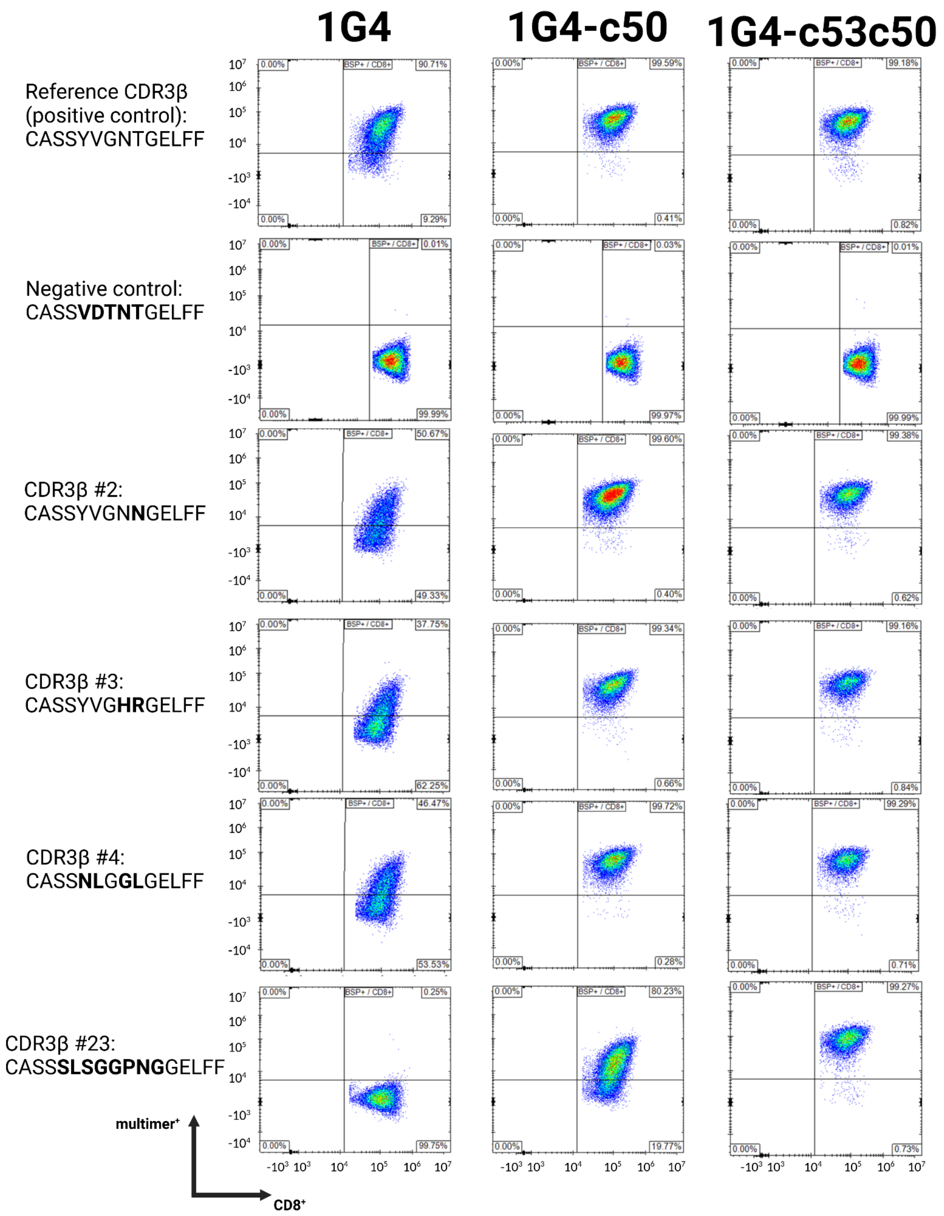

Fig. S5. Representative FACS plots showing multimer staining results for five TCRs with distinct CDR3β sequences. The TCRs were selected from the 30 tested for validation that are listed in Table 2.

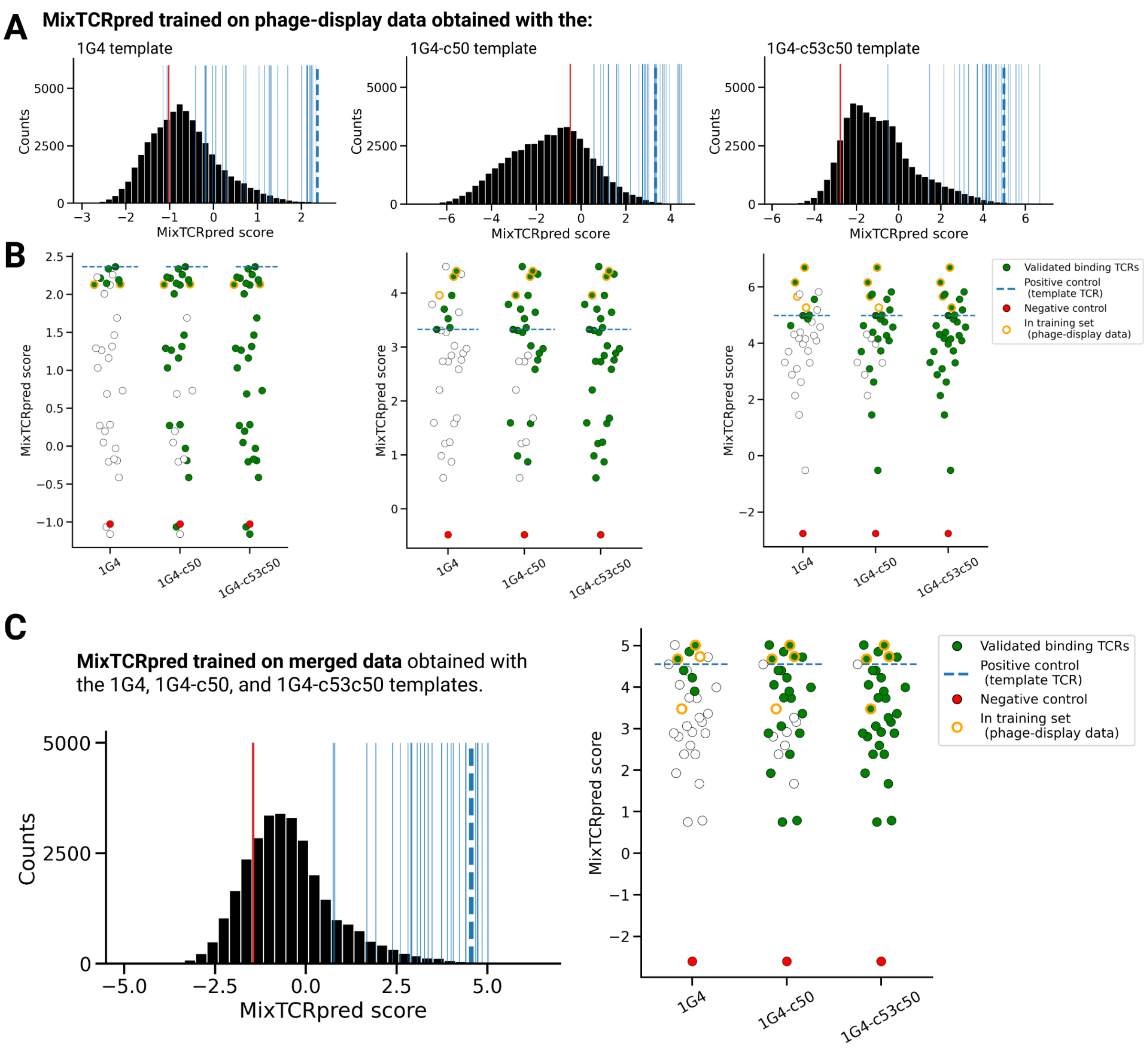

Fig. S6. Results of the MixTCRpred models trained on data from each template separately. (A) Distribution of the MixTCRpred scores. The blue lines show the TCRs selected for experimental testing. The dashed blue line shows the template CDR3β (CASS**YVGNT**GELFF) while the red line shows the negative control (CASS**VDTNT**GELFF). (B) Scores of the CDR3β sequences that could (green) or could not (white) experimentally validated on the 1G4, 1G4-c50, and 1G4-c53c50 templates. The negative control is shown in red. (C) Results of the MixTCRpred models trained on combined data from all three templates.

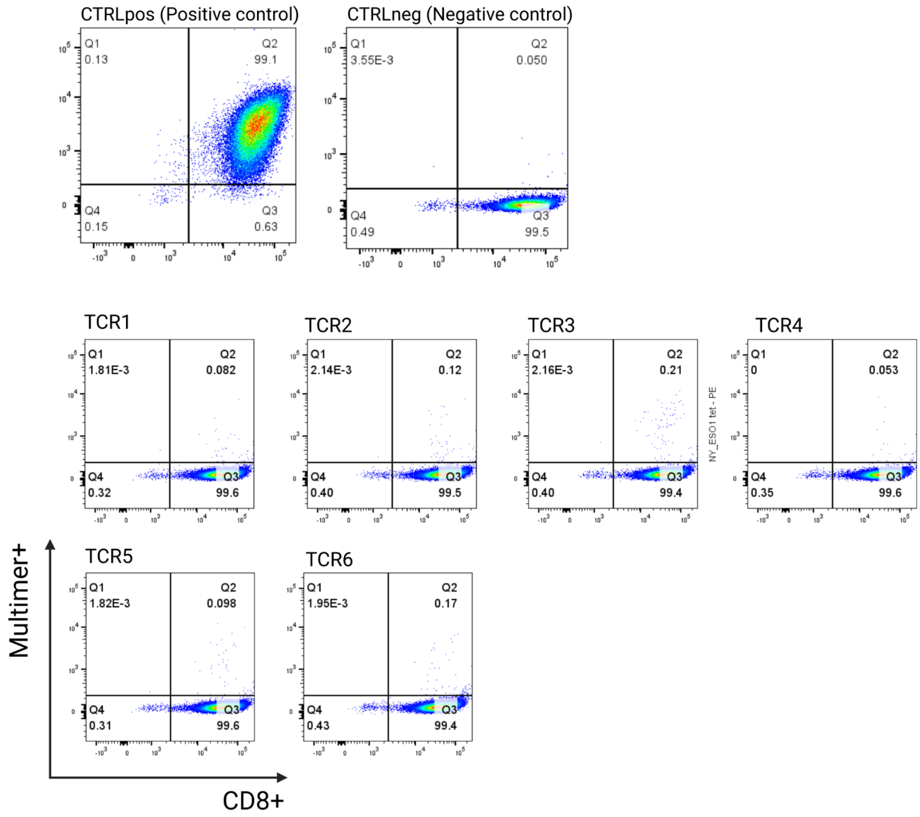

Fig. S7. FACS plots showing the results of multimer staining for six TCRs predicted by MixTCRpred with TRBV or TRBJ genes different from those of the template 1G4 TCR. The TCRs are listed in table S5. The TCRs in Figures S7 and S8 were tested together in the same experiment, using the same positive and negative controls.

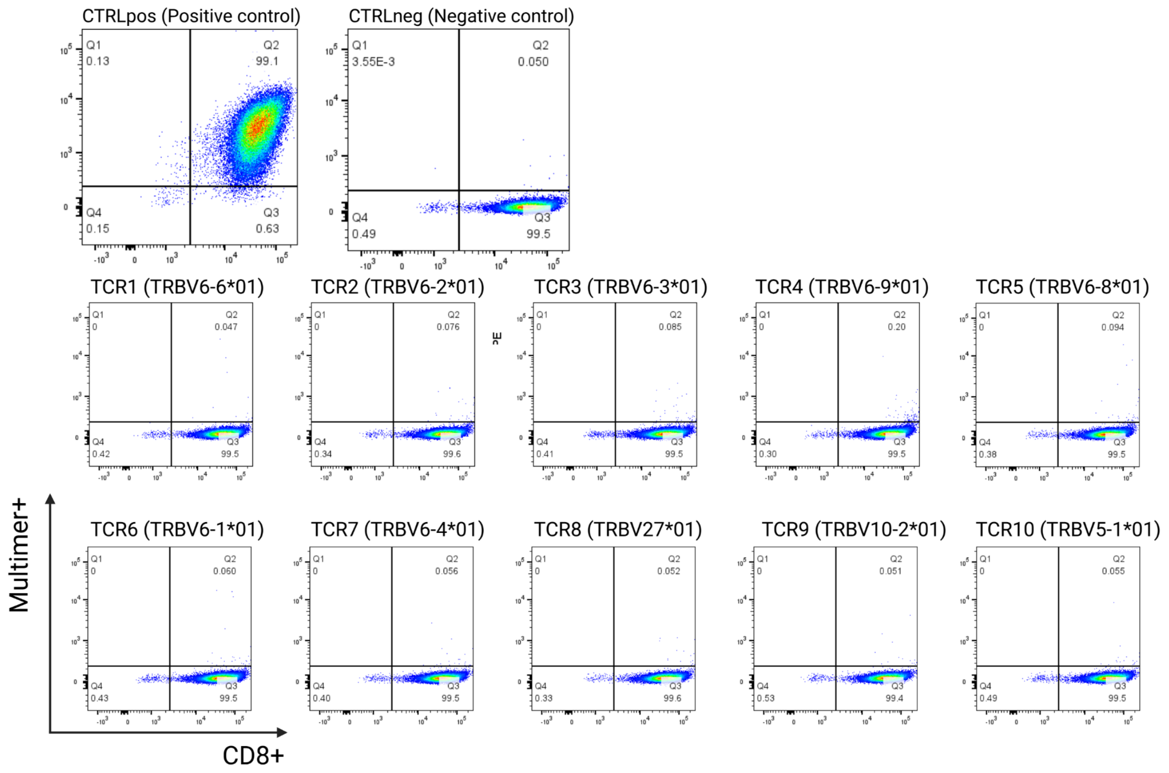

Fig. S8. FACS plots showing the results of multimer staining for ten TCRs with the reference CDR3β (CASSYVGNTGELFF) and J segment (TRBJ2-2*01) and TRBV different from that of the template 1G4 TCR (indicated in parentheses). The TCRs are listed in table S6. The TCRs in Figures S7 and S8 were tested together in the same experiment, using the same positive and negative controls.

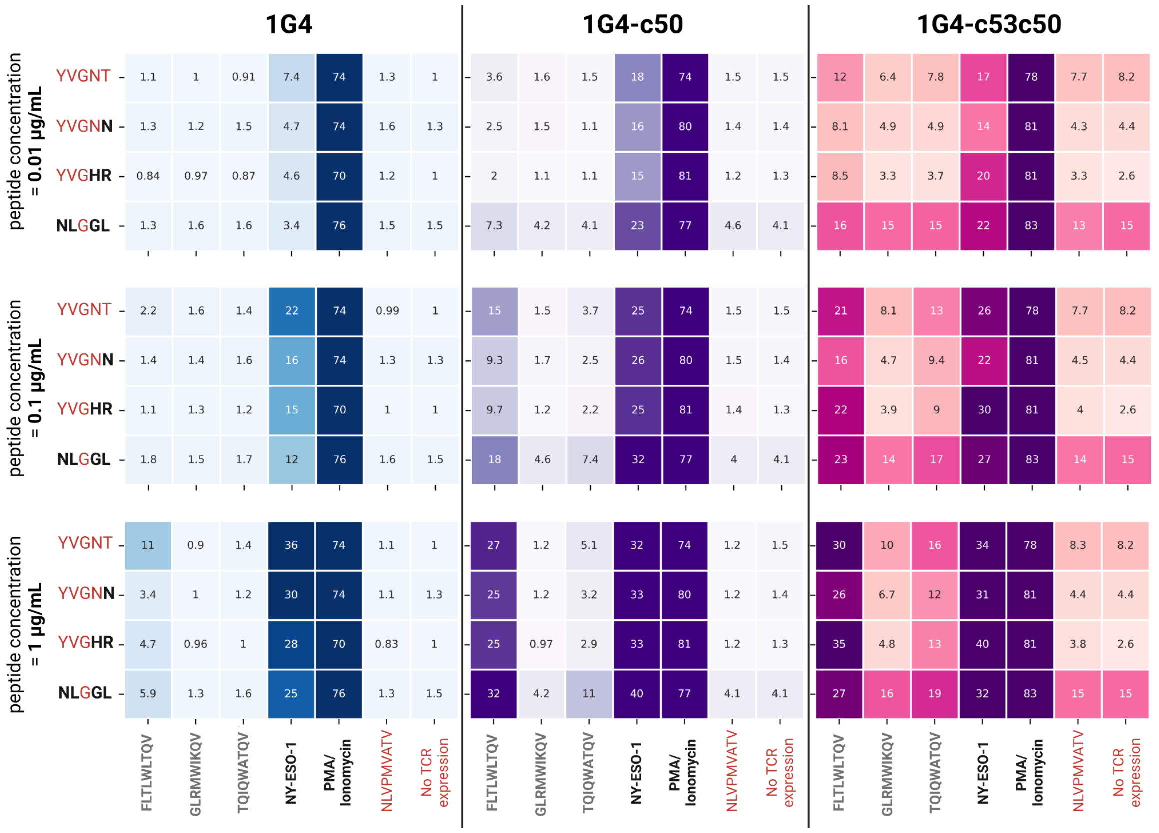

Fig. S9. Heatmaps showing the fraction of CD69+PD-1+ Jurkat cells encoding four TCRs with different CDR3β sequences based on the three template TCRs. Jurkat cells were co-cultured overnight with peptide-pulsed T2 cells, at multiple peptide concentrations (0.01, 0.1, and 1 µg/mL).

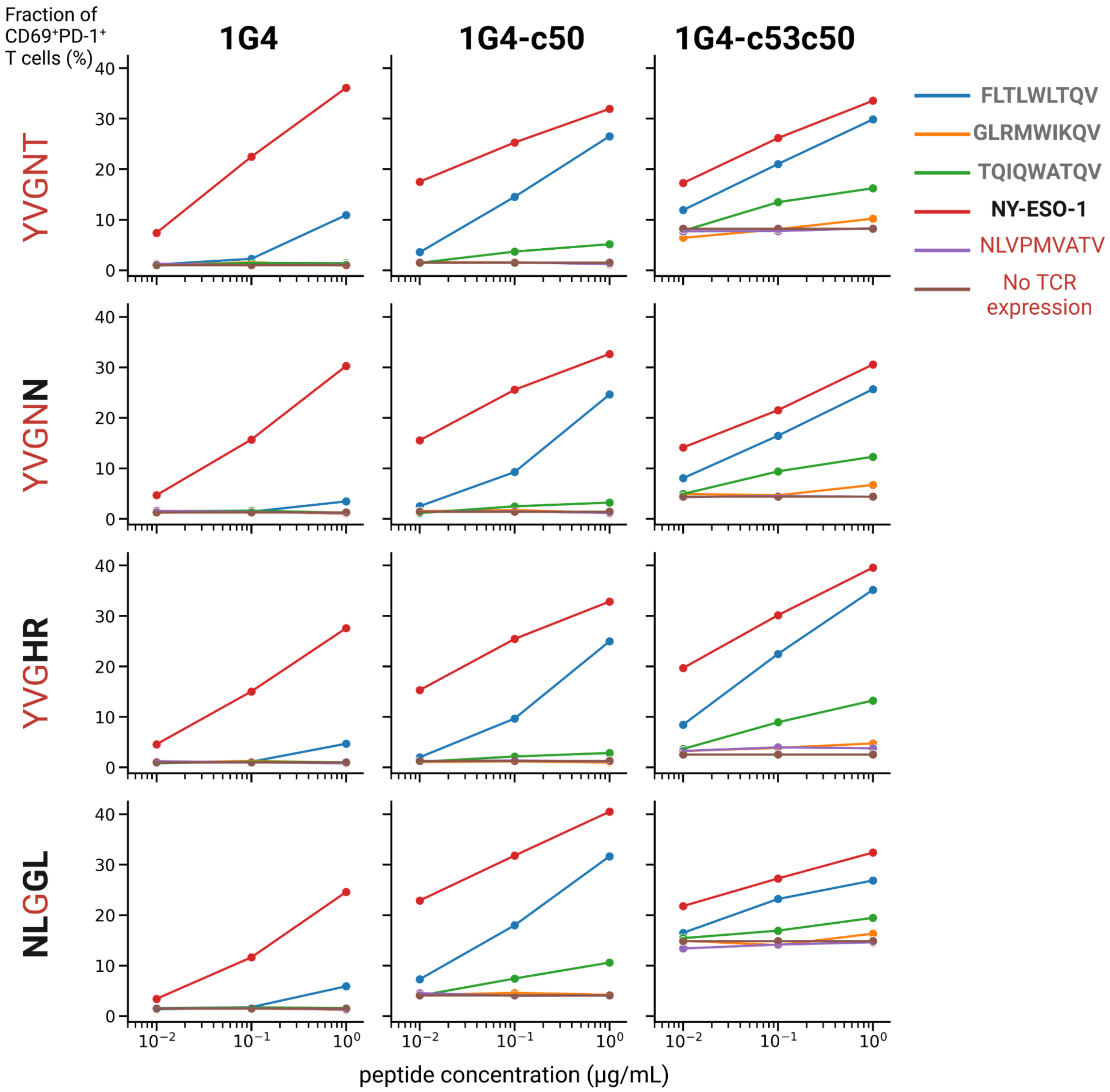

Fig. S10. The fraction of CD69+PD-1+ Jurkat cells expressing four TCRs with distinct CDR3β sequences. Jurkat cells were co-cultured overnight with peptide-pulsed T2 cells at multiple peptide concentrations (0.01, 0.1, and 1 μg/mL).

1G4 WT ESO-1 at 1 μg/mL

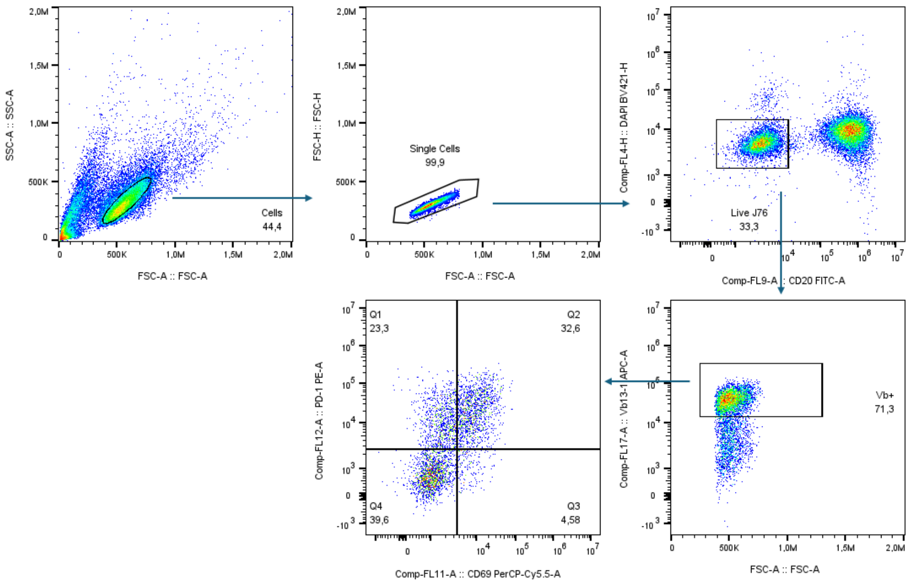

Fig. S11. Illustration of the gating strategy for the co-culture experiments. Live single cells are gated, T cells identified, and PD-1 and CD69 expression measured.

Table S1. Codon mixture of the diversified CDR3β library. The library was devised to approximate the composition of core regions of CDR3β loop in TCR repertoires.

AA		Codon (antisense)	% in mixture
F	Phe	GAA	2
V	Val	AAC	4
D	Asp	ATC	5
H	His	ATG	1
L	Leu	CAG	8
M	Met	CAT	1
R	Arg	TCT	7
Q	Gln	CTG	5
S	Ser	AGA	10
T	Thr	GGT	8
N	Asn	GTT	4
A	Ala	TGC	7
P	Pro	TGG	6
E	Glu	TTC	3
I	Ile	GAT	2
W	Trp	CCA	1
G	Gly	ACC	22
K	Lys	TTT	1
Y	Tyr	GTA	3

Table S2. Primers used in the phage display screening.

Vβ CDR3 library oligos [PCR1]	Sequence
Forward Primer (FR FOR21G4)	5'-CGT GTA CCA GCT GCG TGA TAG C-3'
1G4CDR3_TRIM5_REV	5’-GCC TTC ACC AAA AAA CAG CTC ACC — — — — — GGA GCT GGC ACA GAA GTA CAC-3’
1G4CDR3_TRIM7_REV	5’-GCC TTC ACC AAA AAA CAG CTC ACC — — — — — — — GGA GCT GGC ACA GAA GTA CAC-3’
1G4CDR3_TRIM9_REV	5’-GCC TTC ACC AAA AAA CAG CTC ACC — — — — — — — — — GGA GCT GGC ACA GAA GTA CAC-3’
**Vβ CDR3 library oligos [PCR2]**	**Sequence**
Forward Primer (FR FOR21G4)	5'-CGT GTA CCA GCT GCG TGA TAG C-3'
FR4Xho_REV1G4	5’-GTT CTT AAG GTC CTC GAG TAC GGT CAG GCG AGA GCC TTC ACC AAA AAA CAG CTC ACC-3’
**NGS amplification oligos**	**Sequence**
NGS_FR_FOR21G4	5’-GTC TCG TGG GCT CGG AGA TGT GTA TAA GAG ACA GCG TGT ACC AGC TGC GTG ATA G-3’
NGS_FR4_REV1G4	5’-TCG TCG GCA GCG TCA GAT GTG TAT AAG AGA CAG CTT CAC CAA AAA ACA GCT CAC C-3’

Table S3. Comparison of theoretical diversity and the size of the constructed TCR library for randomized CDR3β regions of varying lengths.

Length of random region	Theoretical diversity	Size of constructed library (number of bacterial clones)
5	2.48×10^{6}	2.7×10^{8}
7	8.94×10^{8}	2.6×10^{8}
9	3.23×10^{11}	6.1×10^{8}

Table S4. List of five randomly selected TCRs that were filtered out using the MoDec algorithm.

TCR number	TRAV	TRAJ	CDR3α	TRBV	TRBJ	CDR3β	% of multimer+CD8+ Jurkat cells
**Positive control (template 1G4)**	TRAV21*01	TRAJ6*01	CAVRPTSGGSYIPTF	TRBV6-5*01	TRBJ2-2*01	CASSYVGNTGELFF	98.90
**Negative control**	TRAV21*01	TRAJ6*01	CAVRPTSGGSYIPTF	TRBV6-5*01	TRBJ2-2*01	CASSVDTNTGELFF	0.080
TCR1	TRAV21*01	TRAJ6*01	CAVRPTSGGSYIPTF	TRBV6-5*01	TRBJ2-2*01	CASSKRWLQGELFF	0.074
TCR2	TRAV21*01	TRAJ6*01	CAVRPTSGGSYIPTF	TRBV6-5*01	TRBJ2-2*01	CASSGYISAGELFF	0.098
TCR3	TRAV21*01	TRAJ6*01	CAVRPTSGGSYIPTF	TRBV6-5*01	TRBJ2-2*01	CASSRNVWYGELFF	0.072
TCR4	TRAV21*01	TRAJ6*01	CAVRPTSGGSYIPTF	TRBV6-5*01	TRBJ2-2*01	CASSDAYSEGELFF	0.084
TCR5	TRAV21*01	TRAJ6*01	CAVRPTSGGSYIPTF	TRBV6-5*01	TRBJ2-2*01	CASSMDLKLINGELFF	0.081

Table S5. Six TCRs with high MixTCRpred score and TRBV and TRBJ segments different from that of the template 1G4 (TRBV6-5*01 and TRBJ2-2*01).

TCR number	TRAV	TRAJ	CDR3α	TRBV	TRBJ	CDR3β	MixTCR pred score	% of multimer+ CD8+ Jurkat cells
**Positive control (template 1G4)**	TRAV21*01	TRAJ6*01	CAVRPTSGGSYIPTF	TRBV6-5*01	TRBJ2-2*01	CASSYVGNTGELFF	4.73	99.1
**Negative control**	TRAV21*01	TRAJ6*01	CAVRPTSGGSYIPTF	TRBV6-5*01	TRBJ2-2*01	CASSVDTNTGELFF	-1.44	0.050
TCR1	TRAV21*01	TRAJ6*01	CAVRPTSGGSYIPTF	**TRBV6-6*04**	TRBJ2-2*01	**CASSHTGSTGELFF**	5.36	0.082
TCR2	TRAV21*01	TRAJ6*01	CAVRPTSGGSYIPTF	**TRBV6-6*04**	TRBJ2-2*01	**CASSPLGGPGELFF**	4.70	0.120
TCR3	TRAV21*01	TRAJ6*01	CAVRPTSGGSYIPTF	**TRBV6-2*01**	TRBJ2-2*01	**CASSNIGHTGELFF**	1.89	0.210
TCR4	TRAV21*01	TRAJ6*01	CAVRPTSGGSYIPTF	TRBV6-5*01	**TRBJ1-4*01**	**CASSHLGGDEKLFF**	3.32	0.053
TCR5	TRAV21*01	TRAJ6*01	CAVRPTSGGSYIPTF	**TRBV6-6*04**	**TRBJ1-1*01**	**CASSPLGGWGEAFF**	3.42	0.098
TCR6	TRAV21*01	TRAJ6*01	CAVRPTSGGSYIPTF	TRBV6-5*01	**TRBJ1-1*01**	**CASSLTGGFGEAFF**	3.04	0.170

Table S6. TCRs with the reference CDR3β sequence (CASSYVGNTGELFF) and V segments closely related to the TRBV6-5*01 of the template 1G4.

	TRAV	TRAJ	CDR3α	TRBV	TRBJ	CDR3β	% of multimer+CD8+ Jurkat cells
**Positive control (template 1G4)**	TRAV21*01	TRAJ6*01	CAVRPTSGGSYIPTF	TRBV6-5*01	TRBJ2-2*01	CASSYVGNTGELFF	99.1
**Negative control-**	TRAV21*01	TRAJ6*01	CAVRPTSGGSYIPTF	TRBV6-5*01	TRBJ2-2*01	CASSVDTNTGELFF	0.050
TCR1	TRAV21*01	TRAJ6*01	CAVRPTSGGSYIPTF	**TRBV6-6*01**	TRBJ2-2*01	CASSYVGNTGELFF	0.047
TCR2	TRAV21*01	TRAJ6*01	CAVRPTSGGSYIPTF	**TRBV6-2*01**	TRBJ2-2*01	CASSYVGNTGELFF	0.076
TCR3	TRAV21*01	TRAJ6*01	CAVRPTSGGSYIPTF	**TRBV6-3*01**	TRBJ2-2*01	CASSYVGNTGELFF	0.085
TCR4	TRAV21*01	TRAJ6*01	CAVRPTSGGSYIPTF	**TRBV6-9*01**	TRBJ2-2*01	CASSYVGNTGELFF	0.200
TCR5	TRAV21*01	TRAJ6*01	CAVRPTSGGSYIPTF	**TRBV6-8*01**	TRBJ2-2*01	CASSYVGNTGELFF	0.094
TCR6	TRAV21*01	TRAJ6*01	CAVRPTSGGSYIPTF	**TRBV6-1*01**	TRBJ2-2*01	CASSYVGNTGELFF	0.060
TCR7	TRAV21*01	TRAJ6*01	CAVRPTSGGSYIPTF	**TRBV6-4*01**	TRBJ2-2*01	CASSYVGNTGELFF	0.056
TCR8	TRAV21*01	TRAJ6*01	CAVRPTSGGSYIPTF	**TRBV27*01**	TRBJ2-2*01	CASSYVGNTGELFF	0.052
TCR9	TRAV21*01	TRAJ6*01	CAVRPTSGGSYIPTF	**TRBV10-2*01**	TRBJ2-2*01	CASSYVGNTGELFF	0.051
TCR10	TRAV21*01	TRAJ6*01	CAVRPTSGGSYIPTF	**TRBV5-1*01**	TRBJ2-2*01	CASSYVGNTGELFF	0.055

Data S1. The randomized CDR3β sequences of the input phage libraries.

Data S2. The CDR3β sequences obtained with the phage display screening after panning with the NY-ESO-1 epitope, and the results of the motif deconvolution.

Data S3. The HLA ligands identified by mass spectrometry from Jurkat cells transduced with the HLA-A*02:01 molecule along with the MixMHCpred results.
